# CD153/CD30 signaling promotes age-dependent tertiary lymphoid tissue expansion and kidney injury

**DOI:** 10.1172/JCI146071

**Published:** 2022-01-18

**Authors:** Yuki Sato, Akiko Oguchi, Yuji Fukushima, Kyoko Masuda, Naoya Toriu, Keisuke Taniguchi, Takahisa Yoshikawa, Xiaotong Cui, Makiko Kondo, Takeshi Hosoi, Shota Komidori, Yoko Shimizu, Harumi Fujita, Li Jiang, Yingyi Kong, Takashi Yamanashi, Jun Seita, Takuya Yamamoto, Shinya Toyokuni, Yoko Hamazaki, Masakazu Hattori, Yasunobu Yoshikai, Peter Boor, Jürgen Floege, Hiroshi Kawamoto, Yasuhiro Murakawa, Nagahiro Minato, Motoko Yanagita

**Affiliations:** 1 Department of Nephrology,; 2 Medical Innovation Center TMK Project, Graduate School of Medicine, Kyoto University, Kyoto, Japan.; 3 RIKEN Center for Integrative Medical Sciences, Yokohama, Kanagawa, Japan.; 4 Department of Immunosenescence and; 5 Department of Immunology and Cell Biology, Graduate School of Medicine, Kyoto University, Kyoto, Japan.; 6 Department of Immunology, Institute for Frontier Medical Science, and; 7 Institute for the Advanced Study of Human Biology (ASHBi), Kyoto University, Kyoto, Japan.; 8 Department of Pathology and Biological Responses, Nagoya University Graduate School of Medicine, Nagoya, Aichi, Japan.; 9 Medical Sciences Innovation Hub Program, RIKEN, Tokyo, Japan.; 10 Department of Life Science Frontiers, Kyoto University, Kyoto, Japan.; 11 Medical-risk Avoidance Based on iPS Cells Team, RIKEN Center for Advanced Intelligence Project (AIP), Kyoto, Japan.; 12 Sydney Medical School, The University of Sydney, New South Wales, Australia.; 13 Laboratory of Immunobiology, Center for iPS Cell Research and Application (CiRA), Kyoto University, Kyoto, Japan.; 14 Division of Host Defense, Network Center for Infectious Disease, Medical Institute of Bioregulation, Kyushu University, Fukuoka, Japan.; 15 Institute of Pathology and; 16 Department of Nephrology, RWTH University of Aachen, Aachen, Germany.; 17 DSK Project, Graduate School of Medicine, Kyoto University, Kyoto, Japan.

**Keywords:** Inflammation, Nephrology, Chronic kidney disease

## Abstract

Tertiary lymphoid tissues (TLTs) facilitate local T and B cell interactions in chronically inflamed organs. However, the cells and molecular pathways that govern TLT formation are poorly defined. Here, we identified TNF superfamily CD153/CD30 signaling between 2 unique age-dependent lymphocyte subpopulations, CD153^+^PD-1^+^CD4^+^ senescence-associated T (SAT) cells and CD30^+^T-bet^+^ age-associated B cells (ABCs), as a driver for TLT expansion. SAT cells, which produced ABC-inducing factors IL-21 and IFN-γ, and ABCs progressively accumulated within TLTs in aged kidneys after injury. Notably, in kidney injury models, CD153 or CD30 deficiency impaired functional SAT cell induction, which resulted in reduced ABC numbers and attenuated TLT formation with improved inflammation, fibrosis, and renal function. Attenuated TLT formation after transplantation of CD153-deficient bone marrow further supported the importance of CD153 in immune cells. Clonal analysis revealed that SAT cells and ABCs in the kidneys arose from both local differentiation and recruitment from the spleen. In the synovium of aged rheumatoid arthritis patients, T peripheral helper/T follicular helper cells and ABCs also expressed CD153 and CD30, respectively. Together, our data reveal a previously unappreciated function of CD153/CD30 signaling in TLT formation and propose targeting the CD153/CD30 signaling pathway as a therapeutic target for slowing kidney disease progression.

## Graphical abstract

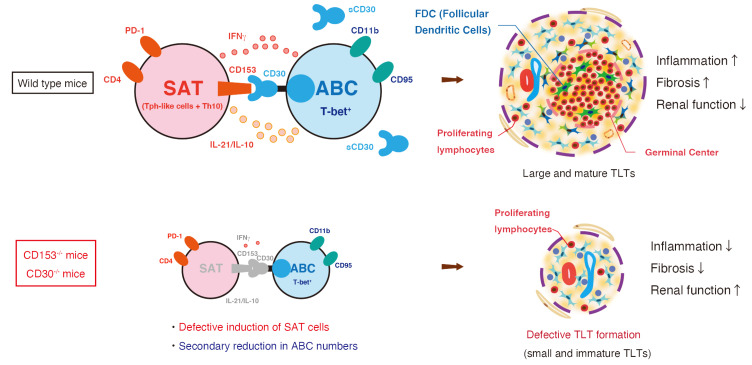

## Introduction

Tertiary lymphoid tissues (TLTs) are inducible ectopic lymphoid tissues that facilitate local T and B cell interactions in chronically inflamed organs (1–3). TLTs initiate antigen-driven immune responses and underlie chronic inflammation. TLTs have been identified in various human diseases, including cancer, infection, and autoimmunity (1–3). Aging has also recently been identified as a predisposing factor for TLT development (4–6).

Acute kidney injury (AKI) is a global public health problem resulting in 1.7 million deaths annually (7). The prognosis of AKI in the elderly is particularly poor and often progresses to chronic kidney disease (CKD) (8, 9). Although the incidence of AKI increases over years, especially in the elderly, no drug has been demonstrated to be effective for preventing CKD progression after AKI (10–12). We previously reported that aged kidneys, but not young kidneys, exhibit TLTs after AKI, which underlie maladaptive repair (4). Furthermore, we demonstrated that TLTs in the kidney develop through several distinct developmental stages irrespective of etiology, and that the developmental progression is associated with the severity of kidney injury in mice and human (13). Although TLTs are also detectable in various kidney diseases (1, 2), the cellular and molecular details of T and B cell interactions within TLTs and signaling pathways governing TLT expansion remain unknown.

Aging affects the functionality and composition of lymphocytes (14), and several age-associated lymphocyte subsets have been identified (15). With aging, unique memory CD4^+^ T cells with the features of cellular senescence, termed “senescence-associated T (SAT) cells,” accumulate progressively in mice (15). SAT cells are CD4^+^ T cells defined by cell-surface expression of PD-1 and CD153 (a TNF superfamily molecule), and secrete humoral factors such as osteopontin (OPN) and sclerostin domain–containing protein 1 (SOSTDC1) (15, 16). Interestingly, thymectomy at the young adult stage leads to accelerated SAT cell generation (17), which is consistent with the observation that T cell aging is accelerated in thymectomized human patients (18) and suggests SAT development is involved in T cell proliferation. Similarly, a rare subset of B cells that express the transcription factor T-bet and CD11b/CD11c accumulates progressively with aging (19–21). These B cells are termed “age-associated B cells (ABCs)” and are of particular interest because they are involved in various pathological conditions by producing antibodies and acting as antigen-presenting cells (19–21). The generation of ABCs is dependent on B cell–activating cytokine IL-21 and direct physical T and B cell interactions (22). Interestingly, SAT cells and ABCs are induced in the same pathological conditions such as autoimmune diseases (16, 23) and obesity (24, 25). However, the relationship between these two unique age-dependent lymphocytes has been unknown.

In the present study, we identified SAT cells and ABCs as key driver cell populations in age-dependent TLT expansion. SAT cells and ABCs gradually accumulated and coexisted within TLTs after injury. SAT cells produced ABC-inducing factors such as IL-21 and IFN-γ. CD153 and its receptor CD30 were specifically expressed in SAT cells and ABCs in aged injured kidneys, respectively. Genetic deficiency of CD153 or CD30 impaired functional SAT cell development, which resulted in decreased numbers of ABCs and attenuated TLT formation, with improved renal function. Mechanistically, CD153/CD30 signaling was indispensable for SAT cells to acquire the capability of ABC induction. These findings identify the CD153-CD30 interaction as a pivotal regulator of age-dependent TLT formation and suggest that targeting CD153/CD30 signaling may be a valuable strategy for the prevention and treatment of kidney diseases in the elderly.

## Results

### SAT cells and ABCs are accumulated in aged injured kidneys with multiple TLTs.

**Figure 1:**
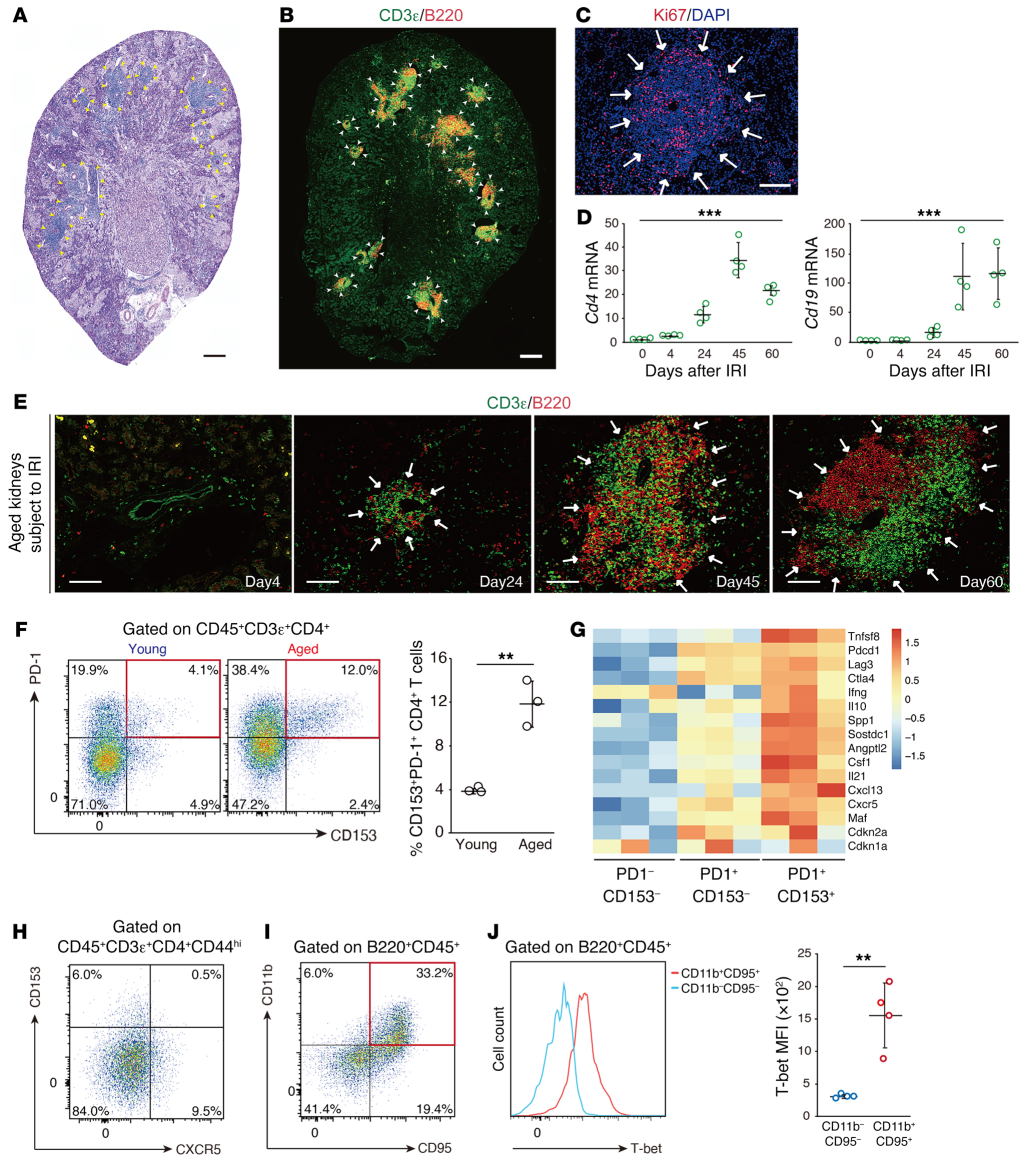
SAT cells and ABCs accumulate in aged injured kidneys with TLTs. Representative images of aged kidneys 45 days after ischemic reperfusion injury (IRI) by (**A**) periodic acid–Schiff staining and immunofluorescent staining of (**B**) CD3ε and B220 and (**C**) Ki67 and DAPI. Arrows and arrowheads indicate the localization of TLTs. (**D**) Expression levels of *Cd4* and *Cd19* mRNA (*n* = 4/time point) in aged mouse kidneys following IRI. (**E**) Immunofluorescence of CD3ε and B220 at various time points after 45-minute IRI. (**F**) Representative FACS plots for identification of SAT cells in young and aged injured kidneys (*n* = 3/group). (**G**) Heatmap generated from bulk RNA-Seq analysis depicting the relative expression of selected SAT cell–related genes in PD1^–^CD153^–^, PD1^+^CD153^–^, and PD1^+^CD153^+^CD44^hi^CD4^+^ T cells in aged injured kidneys (*n* = 3/group). (**H**) Representative FACS plot for the identification of CXCR5^+^ cells in SAT cells (*n* = 3/group). (**I**) Representative FACS plot for the identification of ABCs. (**J**) Representative histogram and the quantitative mean fluorescence intensity (MFI) of T-bet in CD95^–^CD11b^–^ cells and ABCs in aged injured kidneys (*n* = 4/group). Data are presented as mean ± SD. Statistical significance was determined by trend test (**D**) or unpaired, 2-tailed *t* test (**F** and **J**). **P < 0.01; ***P < 0.001. Scale bars: 300 μm (**A** and **B**) and 100 μm (**C** and **E**).

We utilized unilateral ischemic reperfusion injury (IRI) in aged mice as an inducible TLT model (4, 13), which allows us to examine the injured kidneys long after IRI induction because the contralateral kidney compensates renal dysfunction. As in our previous study (13), we defined TLTs as organized lymphocyte clusters with signs of proliferation (Figure 1, A–C, and ref. 26). Total numbers of CD4^+^ T cells and B cells increased following IRI (Figure 1D), and TLTs appeared in the later phases of injury and gradually expanded (Figure 1E and Supplemental Figure 1; supplemental material available online with this article; https://doi.org/10.1172/JCI146071DS1). Based on the observation that age-dependent TLT formation depends on CD4^+^ T cells (4) and T cell proliferation promotes SAT cell development (17), we hypothesized that SAT cells are involved in TLT formation, and found a significant accumulation of SAT cells in aged injured kidneys compared with young injured kidneys (Figure 1F).

To characterize renal SAT cells at the transcriptional level, we next performed bulk RNA sequencing (RNA-Seq) analysis and compared the gene expression patterns of CD4^+^CD44^hi^ T cells from aged injured kidneys that were further separated through flow cytometry into 3 populations based on the expression of PD-1 and CD153 (Figure 1G). Similar to SAT cells in the spleen, the expression of *Spp1* (encoding OPN) and *Sostdc1* was upregulated in renal SAT cells. Additionally, we found that genes related to B cell helper function (including *Il21*, *Cxcl13*, *Maf*, and *Il10*) were also upregulated in renal SAT cells compared with other CD4^+^ T cells (Figure 1G). IL-21 and IL-10 not only promote B cell differentiation but also sustain germinal center (GC) reactions (22, 27–29), which is essential for the selection of high-affinity B cells and the development of long-lived plasma cells and memory B cells. Interestingly, despite some expression of mRNA (Figure 1G), SAT cells in the kidneys lacked cell-surface expression of CXCR5 (Figure 1H), which is a marker of follicular helper T cells (Tfh), a subset of CD4^+^ T cells that are specialized in providing help to B cells within GCs in lymph nodes and spleen (30). Since SAT cells produce IFN-γ and IL-21, both of which are essential for the induction of ABCs (19, 22), we examined the presence of ABCs and found the accumulation of T-bet^+^CD11b^+^CD95^+^ ABCs in aged injured kidneys (Figure 1, I and J).

### scRNA-Seq analysis defines transcriptomic profiles of SAT cells and ABCs in aged injured kidneys.

**Figure 2:**
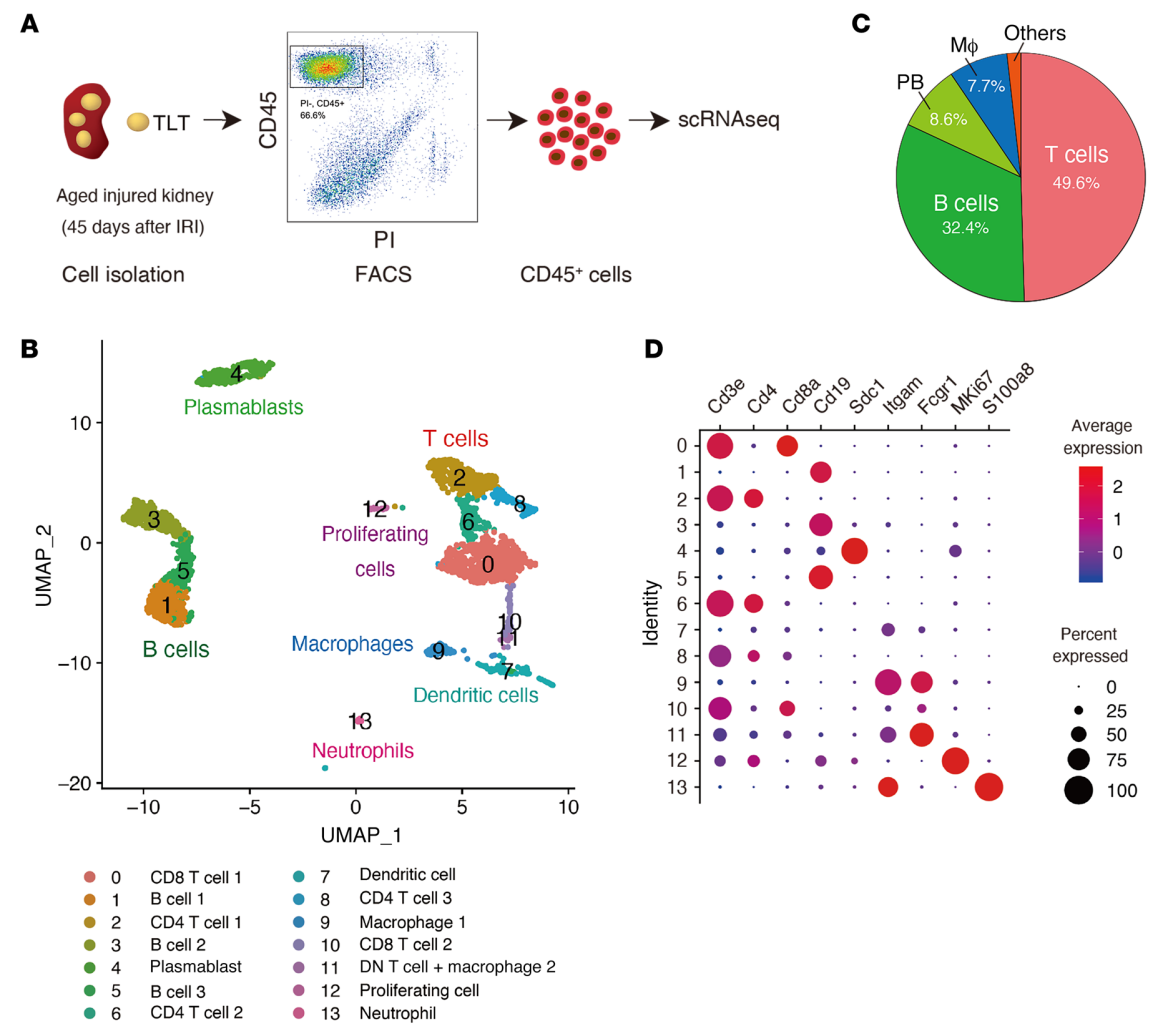
scRNA-Seq analysis of immune cells in aged injured kidneys with multiple TLTs. (**A**) Workflow of the experimental strategy for scRNA-Seq analysis of immune cells from aged injured kidneys with TLTs (*n* = 2). (**B**) UMAP of 2344 CD45^+^ cells from aged injured kidneys with TLTs exhibiting 14 different subsets. (**C**) Pie chart of the relative frequencies of each CD45^+^ cell subset, and (**D**) dot plot showing the expression of canonical marker genes across all 14 subsets. MΦ, macrophage; PB, plasmablast.

To further validate the above results and to unravel the heterogeneity of immune cells in aged injured kidneys, we performed single-cell RNA-Seq (scRNA-Seq) of CD45^+^ immune cells from aged injured kidneys (Figure 2A). After quality control, we retained 2344 CD45^+^ cells with 1943 median genes per cell. Clustering of these cells resulted in 14 clusters (Figure 2B). More than 80% of the cells were lymphocytes (Figure 2C). We annotated the clusters with signatures of known lineage markers, and 12 of the 14 clusters matched established subsets (Figure 2, B and D, and Supplemental Figure 2A): T cells, B cells, plasmablasts, dendritic cells, macrophages, and neutrophils. Cluster 11 was suggested to be a mixture of double-negative T cells (31) and macrophages, and cluster 12 was annotated as proliferating cells (Figure 2B and Supplemental Figure 2, A and B).

**Figure 3:**
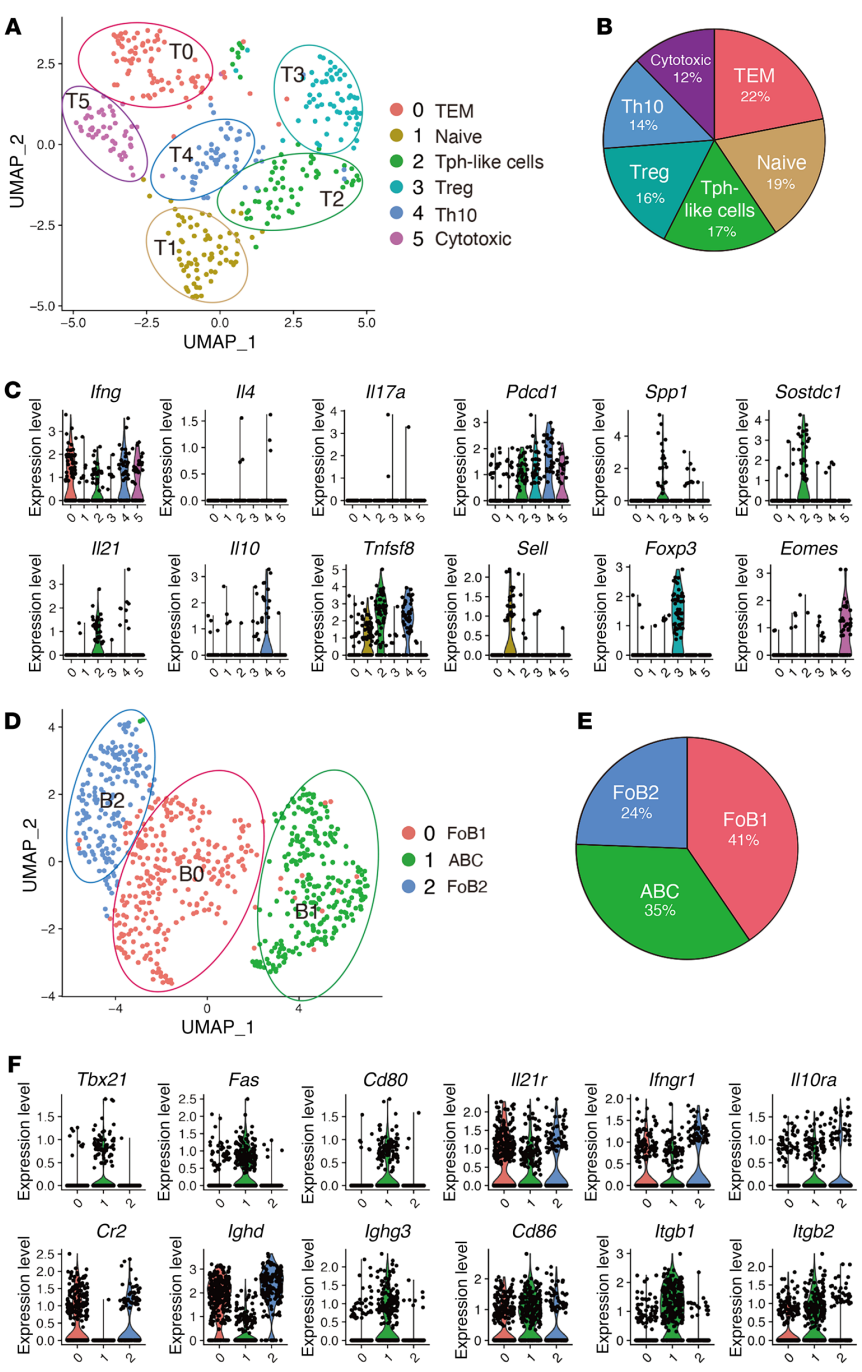
scRNA-Seq analysis defines transcriptomic profiles of SAT cells and ABCs in aged injured kidneys. (**A**–**C**) Focused analysis of CD4^+^ T cells. (**A**) UMAP of 374 CD4^+^ T cells presenting 6 different subsets, (**B**) pie chart of the relative frequencies of each CD4^+^ T cell subset, and (**C**) violin plots showing the expression of 12 marker genes of CD4^+^ T cells. TEM, effector/memory T cells. (**D**–**F**) Focused analysis of B cells. (**D**) UMAP of 684 B cells presenting 3 different subsets. (**E**) Pie chart of the relative frequencies of each B cell subset. (**F**) Violin plots showing the expression of 12 marker genes of B cells. FoB, follicular B cell.

Next, we clustered CD4^+^ T cells with increased resolution, which resulted in 6 distinct subclusters (Figure 3, A and B, and Supplemental Figure 2C). The major proinflammatory cytokine produced by CD4^+^ T cells in aged injured kidneys was IFN-γ (Figure 3C), which was also confirmed by stimulation assay (Supplemental Figure 2D). Based on the established marker gene expression, we annotated effector-memory T (Tem) cells (T0: *Ifng* and *Cxcr6*), naive T cells (T1: *Ccr7* and *Sell*), Treg (T3: *Foxp3* and *Il2ra*), and cytotoxic CD4^+^ T cells (T5: *Eomes* and *Gzmk*). Interestingly, T2 and T4 expressed both *Tnfsf8* and *Pdcd1* (Figure 3C), suggesting that SAT cells were further divided into 2 subpopulations. T1 also expressed *Tnfsf8*, but lacked *Pdcd1* expression (Figure 3C). In addition to *Pdcd1*, T2 and T4 also exhibited higher expression of inhibitory receptors such as *Lag3*, and T2 almost exclusively expressed genes related to humoral factors unique to SAT cells, including *Spp1*, *Sostdc1*, and *Angptl2* (Figure 3C and Supplemental Figure 2C). In addition to *Ifng*, T2 and T4 expressed *Il21* and *Il10* (Figure 3C), respectively. In terms of the B cell helper function and their localization in chronic inflammatory organs, T2 and T4 were reminiscent of IL-21–producing peripheral T helper (Tph) cells and IL-10–producing CD4^+^ T cells (Th10), both of which have been recently identified as CXCR5^–^IFNγ^+^PD-1^hi^CD4^+^ T cell subsets with B cell helper functions in chronically inflamed nonlymphoid organs (32, 33). Despite the high expression of inhibitory receptors such as PD-1, these cells are not globally exhausted but promote B cell responses (32, 33). Therefore, we subsequently refer to these populations as Tph-like cells and Th10.

Within the CD19^+^ B cell compartment, 3 distinct clusters were identified (Figure 3, D and E). Based on the expression of several B cell markers, including *Cr2* and *Tbx21*, we annotated them as 2 types of follicular B cells (FoB1 and FoB2) and ABCs (Figure 3, D–F). Notably, *Ighd* (unswitched IgD^+^) and *Ighg3* (switched IgG^+^) were expressed in FoBs and ABCs, respectively (Figure 3F). Together with the presence of plasmablasts (Figure 2, B–D), aged injured kidneys with TLTs harbored distinct subsets of B cells at different developmental stages, indicating in situ B cell maturation. ABCs expressed higher levels of costimulatory molecule *Cd80* and comparable levels of *Cd86* (Figure 3F), both of which are known to improve ABCs’ ability to present antigen to T cells (20). ABCs also expressed higher levels of *Itgb1* and *Itgb2*, both of which are involved in cell adhesion, and all B cell subpopulations expressed the receptors for IL-21, IFN-γ, and IL-10 (Figure 3F).

### SAT cells and ABCs progressively and correlatively expand within TLTs in aged kidneys after injury.

**Figure 4:**
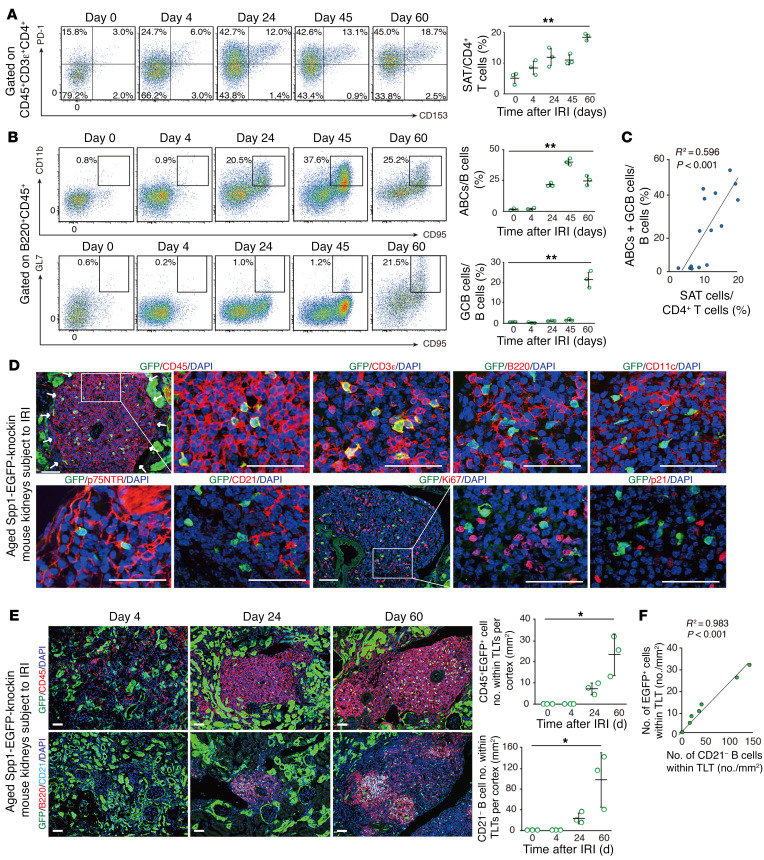
SAT cells and ABCs progressively and correlatively expand within TLTs following kidney injury. (**A** and **B**) Representative FACS plots and quantification of the frequencies of (**A**) SAT cells and (**B**) ABCs and germinal center B cells (GCB cells) in aged kidneys at various time points following ischemic reperfusion injury (IRI). (**C**) The correlation between the frequencies of SAT cells and the sum of the frequencies of ABCs and GCB cells in **A** and **B** (*n* = 3/time point; day 0, 4, 24, 45, and 60). (**D**) Immunofluorescence (IF) of GFP and CD45, CD3ε, B220, CD11c, p75 neurotrophin receptor (p75NTR), CD21, Ki67, and p21 in the kidneys of aged *Spp1*-EGFP-KI mice subjected to IRI. The white arrows indicate the localization of TLTs. Magnified views of the outlined box are shown on the right. (**E**) IF of GFP and CD45; GFP, B220, and CD21; and quantification of the number of CD45^+^GFP^+^ cells and CD21^–^ B cells within TLTs in the renal cortex in serial sections of aged *Spp1*-EGFP-KI mouse kidneys at various time points following IRI (*n* = 3/time point; day 0, 4, 24, and 60). (**F**) The correlation between the number of CD45^+^GFP^+^ cells and CD21^–^ B cells in **E**. Scale bars: 50 μm (**D** and **E**). Data are presented as mean ± SD. Statistical significance was determined by trend test, and correlation was determined by Pearson’s correlation analysis. *P < 0.05; **P < 0.01.

Next, we investigated the longitudinal dynamics of SAT cells and ABCs and their localization in aged kidneys following IRI. While the frequencies of SAT cells in CD4^+^ T cells increased following IRI (Figure 4A), the frequencies of ABCs in B cells peaked on day 45 and declined on day 60 (Figure 4B). Vice versa, GC B cells (GCB cells), defined by the cell-surface expression of CD95 and GL7, dramatically increased on day 60 (Figure 4B). Additionally, the frequencies of SAT cells and CD95^+^ cells, defined as ABCs and GCB cells, in aged injured kidneys exhibited a positive correlation (Figure 4C).

To visualize SAT cells, especially Tph-like cells, we utilized aged *Spp1*-EGFP–knockin (*Spp1*-EGFP-KI) mice (Figure 4, D and E, and refs. 16, 25). Under physiological conditions, *Spp1* was expressed in renal distal tubule epithelial cells (Supplemental Figure 3). In aged *Spp1*-EGFP-KI mouse kidneys subjected to IRI, CD45^+^GFP^+^ cells were detectable almost exclusively within TLTs and colocalized with CD3ε, but not with other hematopoietic cell lineage or stromal cell markers (Figure 4D). Additionally, most of the GFP^+^ cells within TLTs were negative for Ki67 and p21 (Figure 4D). We counted the absolute number of CD45^+^GFP^+^ cells and CD21^–^ B cells, a surrogate for ABCs, within TLTs (Supplemental Figure 4), utilizing serial sections of aged *Spp1*-EGFP-KI mouse kidneys subjected to IRI. We found that both cell types appeared with TLT formation, and their numbers increased over time after TLT development (Figure 4E) and exhibited a significant positive correlation (Figure 4F). Collectively, the spatiotemporally synchronized development of SAT cells and ABCs within TLTs suggests the possibility that the development of these 2 unique age-dependent lymphocyte populations is mechanistically linked.

### CD153/CD30 signaling pathway is identified as the interacting molecules between SAT cells and ABCs in aged injured kidneys.

**Figure 5:**
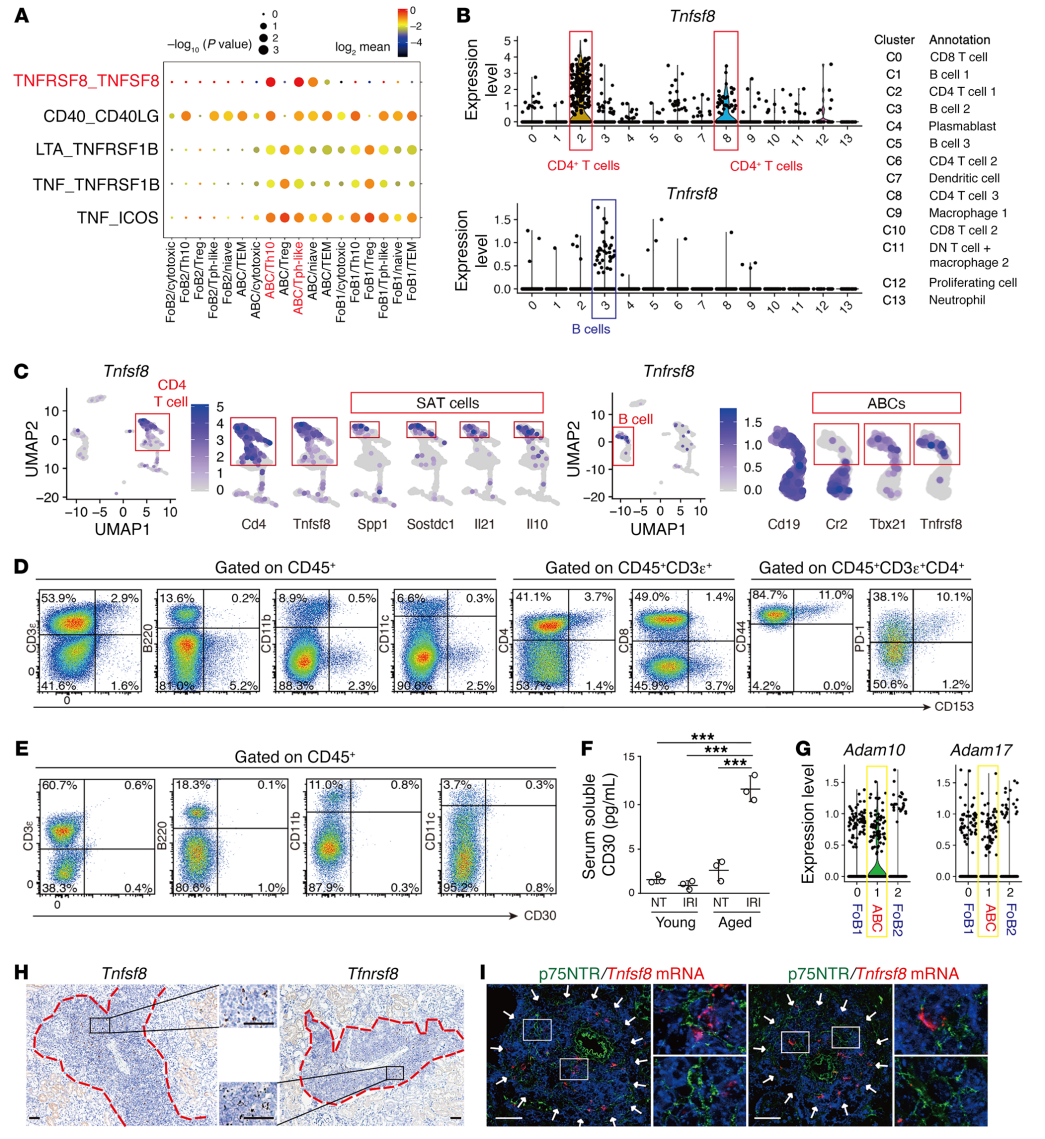
TNF superfamily members CD153 and CD30 are specifically expressed in SAT cells and ABCs, respectively, inside of TLTs in aged injured kidneys. (**A**) Dot plot of ligand-receptor interactions related to TNF superfamily members between 6 CD4^+^ T cell subpopulations and 3 B cell subpopulations identified by scRNA-Seq (see Figure 3). (**B**) Violin plots showing the expression of *Tnfsf8* and *Tnfrsf8* in all CD45^+^ cells. The putative identities of each cluster are specified in the box. DN, double-negative. (**C**) UMAP plot of *Tnfsf8* and *Tnfrsf8* in CD45^+^ cells in Figure 2B and enlargement of those of *Cd4*, *Tnfsf8*, *Spp1*, *Sostdc1*, *Il21*, and *Il10*, and those of *Cd19*, *Cr2*, *Tbx21*, and *Tnfrsf8*. (**D**) Representative FACS plots of CD153 expression on T cells, B cells, macrophages, dendritic cells, CD4^+^ T cells, CD8^+^ T cells, CD44^hi^CD4^+^ T cells, and PD-1^+^CD4^+^ T cells (*n* = 3/group). (**E**) Representative FACS plots of CD30 expression on T cells, B cells, macrophages, and dendritic cells (*n* = 2/group). (**F**) Plasma soluble CD30 (sCD30) levels of young and aged mice with or without (not treated, NT) ischemic reperfusion injury (IRI) induction (*n* = 3/group, samples were collected 45 days after IRI). Data are presented as mean ± SD. Statistical significance was determined by 1-way ANOVA with Bonferroni’s post hoc analysis. ***P < 0.001. (**G**) Violin plots showing the expression of *Adam10* and *Adam17* among 3 B cell subsets identified by scRNA-Seq. (**H**) Representative ISH images of *Tnfsf8* and *Tnfrsf8* in aged kidneys 45 days after IRI, and (**I**) ISH with immunofluorescent costaining for p75NTR, a TLT-associated fibroblast marker, of the aged kidneys. The red dashed lines in **H** and white arrows in **I** indicate the localization of TLTs. A magnified view of the outlined box is shown in the middle in **H** and in the right in **I**. Scale bars: 50 μm.

To predict the potential cellular interactions involved in TLT formation, we performed an unbiased ligand-receptor interaction analysis between CD4^+^ T cell and B cell populations utilizing CellPhoneDB (34). In particular, we focused on TNF superfamily (TNFSF) members because these molecules are crucial in lymphoid tissue development (35). Strikingly, the TNFSF8 (CD153)/TNF receptor superfamily member 8 (TNFRSF8, CD30) pathway was detected as one of the specific interaction molecules between SAT cells (Tph-like cells and Th10) and ABCs (Figure 5A). scRNA-Seq analysis of hematopoietic cells revealed that *Tnfsf8* and *Tnfrsf8* expression was almost exclusively confined to CD4^+^ T cells and B cells, respectively, and was nearly undetectable in other lineage cells (Figure 5B). Among them, *Tnfsf8* and *Tnfrsf8* were expressed almost exclusively in SAT cells and ABCs (Figure 5C). Consistently, cell-surface CD153 was almost exclusively confined to SAT cells (Figure 5D), though cell-surface CD30 was nearly undetectable in any immune cells (Figure 5E), consistent with previous studies from others (36). Since the cell-surface-expressed CD30 is quickly lost after activation because of the shedding by a disintegrin metalloproteinase 10 (ADAM10) and ADAM17 (37, 38), we examined the serum levels of soluble CD30 (sCD30) as well as the expression levels of ADAM10 and ADAM17 in ABCs. Serum sCD30 in aged mice with renal TLTs was significantly increased compared with those of control mice without TLTs (Figure 5F), and *Adam10* as well as *Adam17* were expressed in ABCs (Figure 5G). In situ hybridization (ISH) showed that both *Tnfsf8* (CD153) and *Tnfrsf8* (CD30) expression was almost completely restricted to TLTs (Figure 5H and Supplemental Figure 5), and the signals did not colocalize with those of p75NTR (Figure 5I), a fibroblast marker within TLTs (4). *Tnfrsf8* expression was undetectable before TLT development (Supplemental Figure 6). These data suggest that the expression of CD153 and CD30 was almost completely confined to SAT cells and ABCs, respectively, and further confirm that SAT cells and ABCs coexisted within TLTs.

### CD153 is indispensable for functional SAT cell induction and age-dependent TLT formation, but not for ABC phenotype.

**Figure 6:**
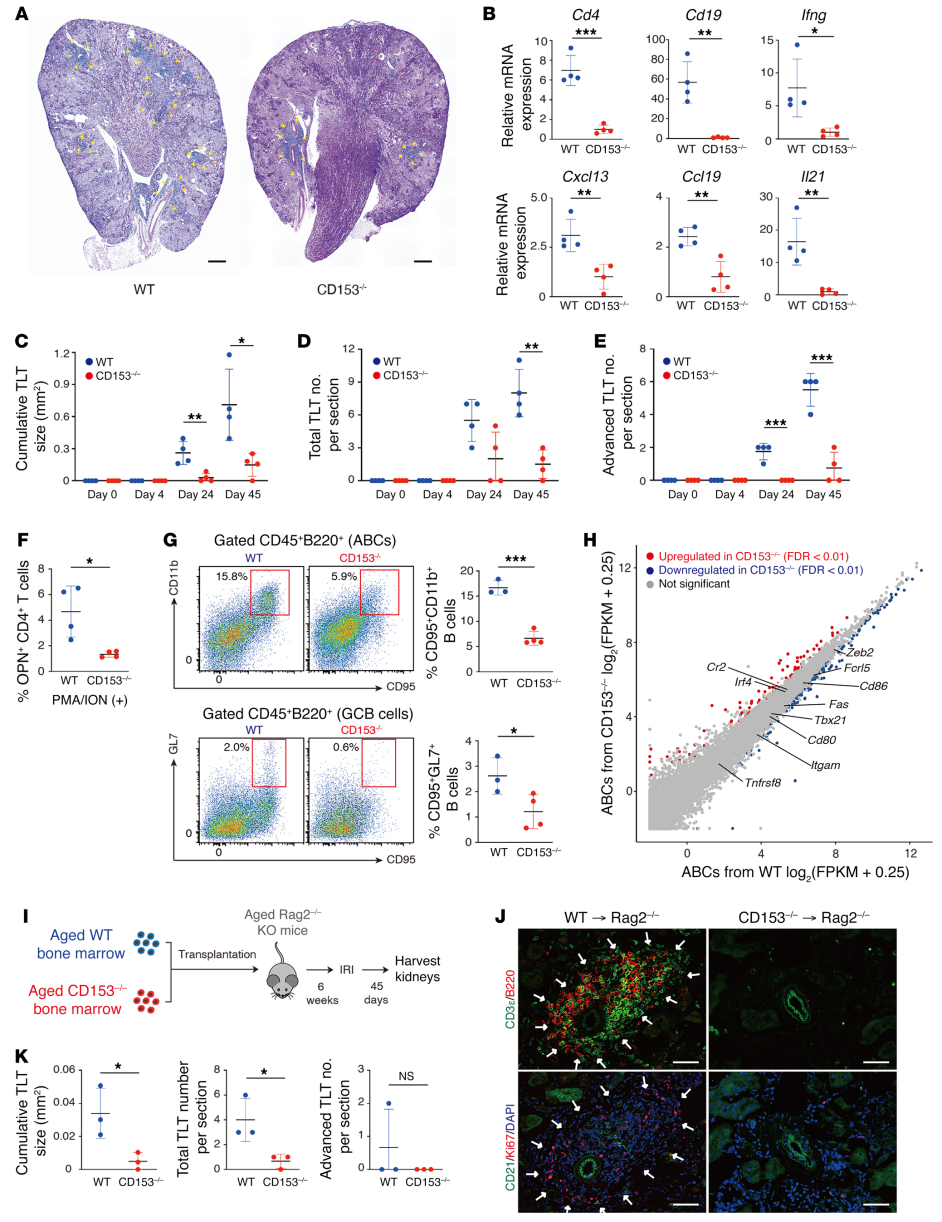
CD153 is essential for functional SAT cell induction and TLT formation. Analysis of aged CD153-deficient (*CD153^–/–^*) mouse kidneys subjected to ischemic reperfusion injury (IRI). (**A**) Periodic acid–Schiff staining and (**B**) *Cd4*, *Cd19, Ifng*, *Cxcl13*, *Ccl19*, and *Il21* mRNA levels (*n* = 4/group) in WT and *CD153^–/–^* mice 45 days after IRI. (**C**) Cumulative TLT sizes per cortex, and the number of (**D**) total and (**E**) advanced-stage TLTs following IRI induction (*n* = 4/group). (**F**) OPN production by CD4^+^ T cells from aged kidneys of WT and *CD153^–/–^* mice 45 days after IRI (*n* = 4/group). Cells were stimulated in bulk culture with PMA and ionomycin. (**G**) Representative FACS plots and frequencies of ABCs and germinal center B (GCB) cells in WT and *CD153^–/–^* mice (*n* = 3–4/group). (**H**) Scatter plot comparing normalized RNA-Seq read counts of ABCs from WT and *CD153^–/–^* mouse kidneys 45 days after IRI (*n* = 3/group). Colored dots indicate transcripts with FDR < 0.01. Red and blue dots indicate genes that are significantly upregulated and downregulated, respectively. (**I**) Experimental protocol of bone marrow transplantation experiments. Bone marrow cells from aged WT and *CD153^–/–^* mice were transplanted into lethally irradiated, aged Rag2-deficient (*Rag2^–/–^*) mice. After a 6-week recovery period, mice were subjected to IRI and sacrificed on day 45. (**J**) Immunofluorescence of CD3ε and B220; and CD21, Ki67, and DAPI. (**K**) Cumulative TLT sizes per cortex and the number of total and advanced-stage TLTs per section (*n* = 3/group). The arrowheads in **A** and arrows in **J** indicate the localization of TLTs. Scale bars: 300 μm (**A**) and 50 μm (**J**). Data are presented as mean ± SD, and statistical significance was determined by an unpaired, 2-tailed *t* test. *P < 0.05; **P < 0.01; ***P < 0.001. NS, not significant.

We next asked whether CD153/CD30 signaling promotes TLT formation utilizing CD153-deficient (*CD153^–/–^*) and CD30-deficient (*CD30^–/–^*) mice. At baseline, both mice exhibited normal kidney structures and function (Supplemental Figure 7, A–E). First, we induced the IRI model in aged *CD153^–/–^* mice. Forty-five days after IRI, *CD153^–/–^* mice exhibited smaller and fewer total TLTs (Figure 6A), and exhibited reduced expression of TLT-associated cytokines, chemokines, as well as *Cd4* and *Cd19* in the kidneys compared with those of wild-type (WT) controls (Figure 6B). Time-course analysis also revealed that *CD153^–/–^* mice exhibited smaller TLT sizes, and fewer total and advanced-stage TLTs at each time point (advanced TLTs are defined as TLTs with follicular dendritic cells; ref. 13 and Figure 6, C–E).

Next, we examined OPN production in CD4^+^ T cells derived from injured kidneys of aged WT mice and *CD153^–/–^* mice. SAT cells secrete OPN in response to T cell receptor (TCR) stimulation (16). We found that CD4^+^ T cells of aged *CD153^–/–^* mouse kidneys exhibited blunted OPN production ability (Figure 6F). Together with decreased expression of the SAT cell–specific gene *Il21* (Figure 6B), these results indicate that SAT cell functions were disturbed in *CD153^–/–^* mice. Consistently, besides the reduction in the total number of T and B lymphocytes (Figure 6B), the frequencies of ABCs and GCB cells in aged injured kidneys of *CD153^–/–^* mice were also reduced (Figure 6G).

We next compared gene expression patterns of ABCs in aged injured kidneys of WT and *CD153^–/–^* mice with bulk RNA-Seq analysis. A total of 402 genes was differentially expressed between these 2 groups (false discovery rate [FDR] < 0.01). The expression of ABC marker genes, including *Tbx21* (the gene encoding T-bet), *Zeb2* (the downstream gene of T-bet), *Fas*, *Itgam*, *Cr2*, and *Fcrl5* (20, 21, 28), as well as genes related to antigen presentation such as *Cd80* and *Cd86* (20, 21, 28) in *CD153^–/–^* mice was similar to those in WT mice (Figure 6H).

Next, to investigate the role of CD153 in immune cells, we performed bone marrow transplantation experiments utilizing Rag2-deficient (*Rag2^–/–^*) mice. After 6 weeks of recovery, aged *Rag2^–/–^* mice were subjected to IRI (Figure 6I). Forty-five days after IRI induction, TLT formation was attenuated in aged *Rag2^–/–^* mice receiving *CD153^–/–^* bone marrow compared with those receiving WT bone marrow (Figure 6, J and K). These results indicate that CD153 expression by immune cells, especially CD4^+^ T cells, is essential for TLT expansion.

### CD30 is also indispensable for functional SAT cell induction and age-dependent TLT formation, but not for ABC phenotype.

**Figure 7:**
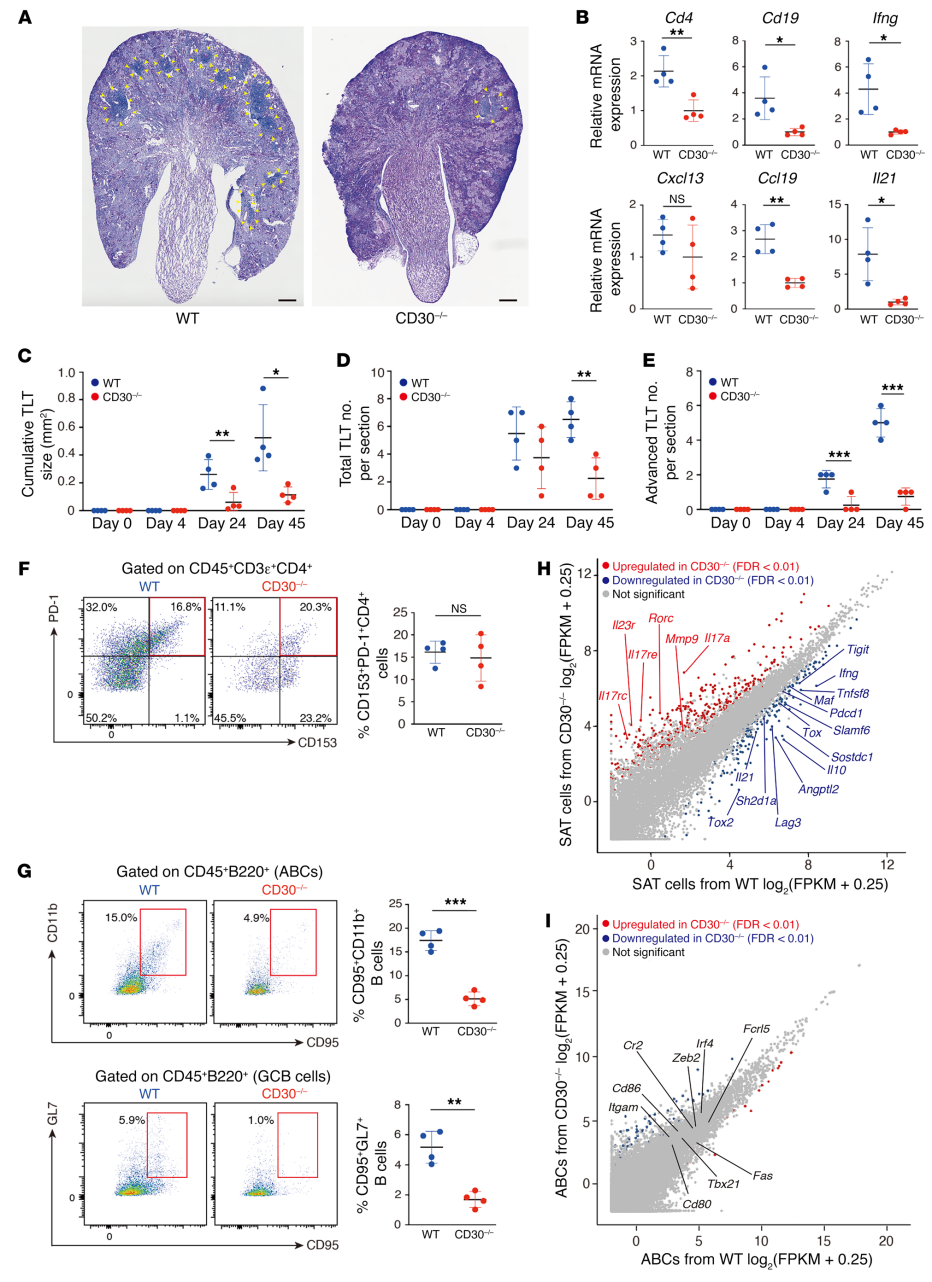
CD30 is also essential for functional SAT cell induction and TLT formation. Analysis of aged CD30-deficient (*CD30^–/–^*) mouse kidneys subjected to IRI. (**A**) Periodic acid–Schiff (PAS) staining and (**B**) *Cd4*, *Cd19*, *Ifng*, *Cxcl13*, *Ccl19*, and *Il21* mRNA levels (*n* = 4/group) in WT and *CD30^–/–^* mice 45 days after ischemic reperfusion injury (IRI). The yellow arrowheads indicate the localization of TLTs. Scale bars: 300 μm. (**C**) Cumulative TLT size per cortex, and the number of (**D**) total and (**E**) advanced-stage TLTs following IRI induction (*n* = 4/group). (**F**) Representative FACS plots and frequencies of SAT cells (*n* = 4), and (**G**) those of ABCs and germinal center B (GCB) cells in WT and *CD30^–/–^* mice (*n* = 4/group). (**H** and **I**) Scatter plots comparing normalized RNA-Seq read counts of (**H**) SAT cells and (**I**) ABCs from WT and *CD30^–/–^* mouse kidneys 45 days after IRI (*n* = 3 per group). Colored dots indicate transcripts with FDR < 0.01. Red and blue dots indicate genes that are significantly upregulated and downregulated, respectively. Data are presented as mean ± SD, and statistical significance was determined by an unpaired, 2-tailed *t* test. *P < 0.05; **P < 0.01; ***P < 0.001. NS, not significant.

We also examined the phenotype of aged *CD30^–/–^* mouse kidneys subjected to IRI, and again found attenuated TLT formation and downregulation of TLT-associated cytokines and chemokines in the kidneys (Figure 7, A–E), though the impact of CD30 deficiency was less profound than that in CD153 deficiency. While total lymphocyte numbers were reduced in the kidneys of aged *CD30^–/–^* mice (Figure 7B), these mice exhibited comparable frequencies of SAT cells in IRI kidneys (Figure 7F). In contrast, similar to those in *CD153^–/–^* mice, the frequencies of ABCs and GCB cells in aged injured kidneys of *CD30^–/–^* mice were reduced (Figure 7G).

To examine how CD153/CD30 signaling impacts the phenotypes of SAT cells and ABCs, we sorted SAT cells and ABCs from aged IRI kidneys of WT and *CD30^–/–^* mice and compared their gene expression patterns with bulk RNA-Seq analysis. A total of 648 and 137 genes were differentially expressed compared with WT, respectively (FDR < 0.01). Notably, SAT cells from *CD30^–/–^* mice exhibited decreased expression levels of *Maf* and *Tox*, key transcriptional factors regulating Tph cells (ref. 39 and Figure 7H). Consistently, the expression levels of *Il21* and *Il10*, which are essential for ABC induction and GCB cell maintenance, as well as those of inhibitory receptors, such as *Pdcd1*, *Lag*, and *Tigit*, which are positively regulated by Tox (40), were dramatically downregulated in SAT cells from *CD30^–/–^* mice (Figure 7H). Additionally, genes for humoral factors unique to SAT cells such as *Sostdc1* and *Angptl2* were also downregulated in *CD30^–/–^* mice. Unexpectedly, SAT cells from *CD30^–/–^* mice expressed higher levels of Th17-related genes such as *Il17a* and *Mmp9* (Figure 7H). On the other hand, the expression levels of ABC marker genes in *CD30^–/–^* mice were similar to those in WT mice (Figure 7I). Together with the results of *CD153^–/–^* mice, these results show that CD153/CD30 signaling is indispensable for several effector functions of SAT cells, such as ABC induction, but not for maintenance of ABC phenotypes.

### Aged CD153^–/–^ mice exhibited improved kidney functions with attenuated TLT formation in an adenine nephropathy model.

**Figure 8:**
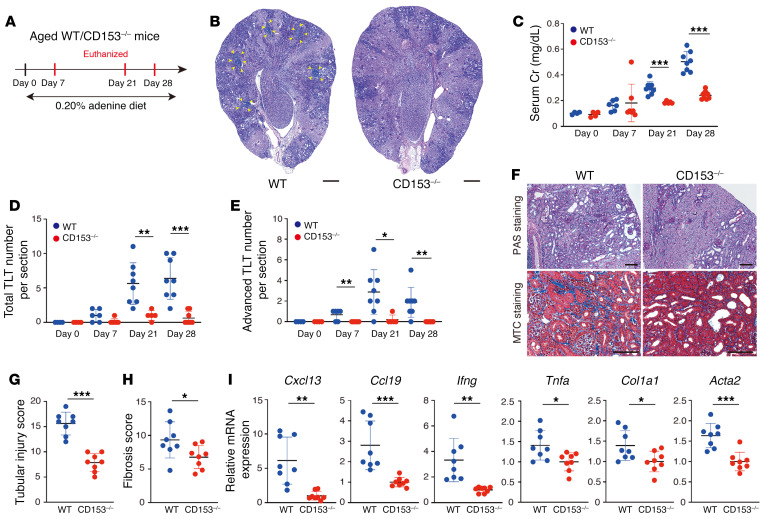
Aged *CD153^–/–^* mice exhibit attenuated TLT formation with improved inflammation, fibrosis, and renal function in adenine nephropathy model. (**A**) Experimental protocol for **B**–**I** (*n* = 4–9 at each time point). In 0.20% adenine nephropathy models, CD153-deficient (*CD153^–/–^*) mice exhibited defective TLT formation and attenuated renal dysfunction, inflammation, and fibrosis, as shown by (**B**) periodic acid–Schiff (PAS) staining, (**C**) serum creatinine (sCr) levels, (**D**) total TLT numbers, (**E**) advanced-stage TLT numbers, (**F**) PAS and Masson’s trichrome (MTC) staining, (**G**) tubular injury scores, (**H**) fibrosis scores, and (**I**) *Cxcl13*, *Ccl19*, *Ifng*, *Tnfa*, *Col1a1*, and *Acta2* mRNA levels. Data are presented as mean ± SD. The yellow arrowheads in **B** indicate the localization of TLTs. Statistical significance was determined by an unpaired, 2-tailed *t* test. *P < 0.05; **P < 0.01; ***P < 0.001. Scale bars: 300 μm (**B**) and 100 μm (**F**).

Mice subjected to unilateral IRI do not exhibit renal dysfunction because the contralateral healthy kidney compensates for the loss of renal function in the injured kidney. Additionally, most of the aged mice do not survive the acute phase in bilateral IRI even under milder ischemic damage because of renal dysfunction (41). Therefore, to evaluate the impact of TLT formation on renal function, we employed an alternative kidney injury model, adenine nephropathy, in aged *CD153^–/–^* mice (Figure 8A). In this model, TLTs appeared 7 days after the initiation of the adenine diet, and the total and advanced TLT numbers gradually increased in parallel with the progression of renal dysfunction (Figure 8, B–E). *CD153^–/–^* mice exhibited significantly smaller TLTs and milder renal dysfunction compared with control mice (Figure 8, C–E). Of note, the difference in renal dysfunction between control and *CD153^–/–^* mice became apparent after the initiation of TLT formation (Figure 8, C–E). Tubular injury, fibrosis scores, and gene expression levels of TLT-associated chemokines and cytokines as well as fibrosis markers were also decreased in *CD153^–/–^* mice on day 28 of adenine nephropathy (Figure 8, F–I).

### SAT cells and ABCs in aged injured kidneys might be derived from both local development and recruitment from the spleen.

**Figure 9:**
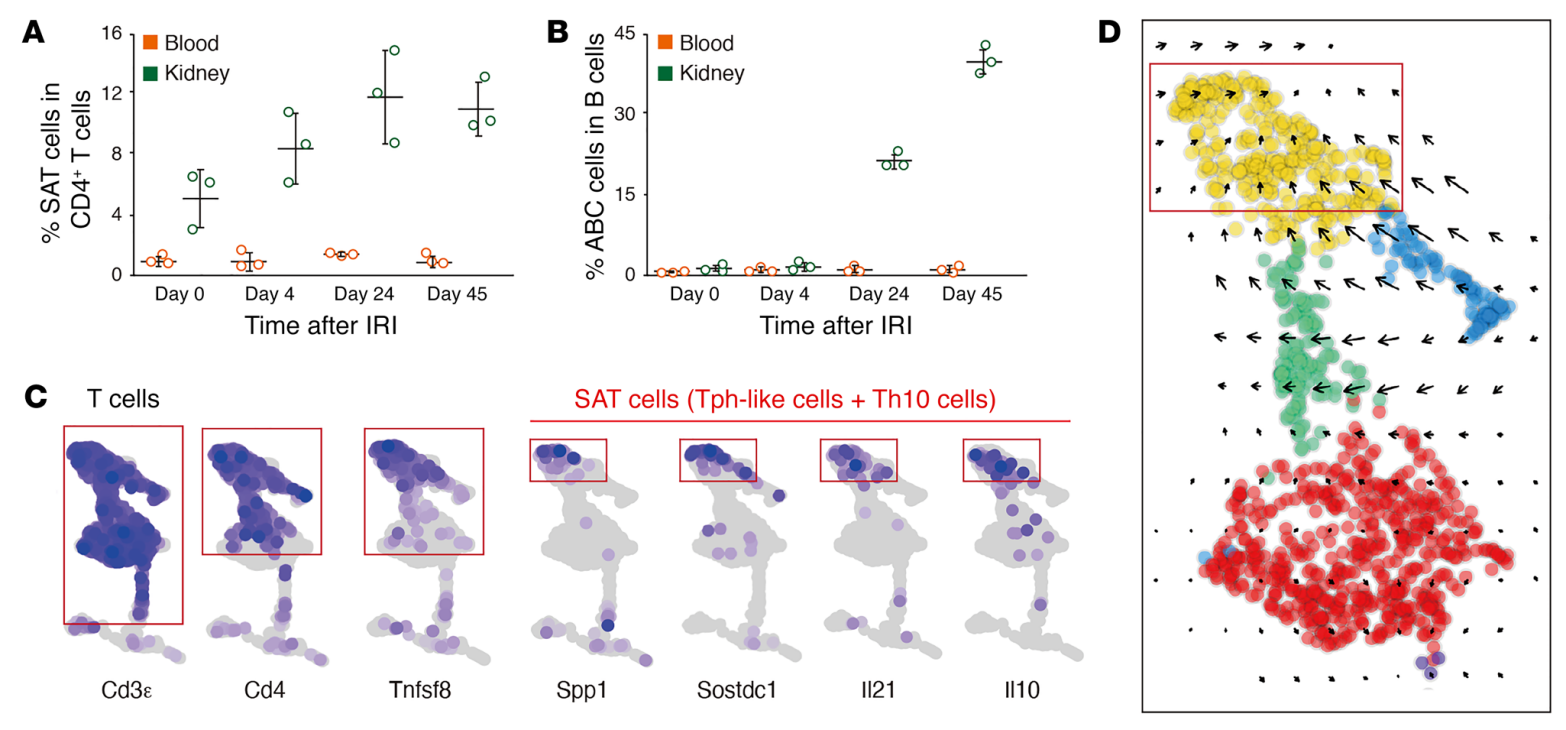
Velocity analysis suggests the local generation of SAT cells in aged injured kidney. (**A **and** B**) Quantification of the frequencies of SAT cells and ABCs in peripheral blood and the kidney at various time points following ischemic reperfusion injury (IRI) (*n* = 3/time points) (Figure 4, A and B are presented again for reference). Data are presented as mean ± SD. (**C**) UMAP plot of *Cd3ε*, *Cd4*, *Tnfsf8*, *Spp1*, *Sostdc1*, *Il21*, and *Il10* (Figure 5C is presented again for reference). (**D**) Velocity force field indicating the average differentiation trajectories (velocity) for cells located in different parts of the UMAP plot.

Next, we asked where SAT cells and ABCs originated from. Unlike aged injured kidneys, the frequencies of SAT cells and ABCs were low in peripheral blood of aged mice following IRI (Figure 4, A and B, and Figure 9, A and B). To test the possibility of local development of SAT cells from other CD4^+^ T cells, we performed velocity analyses of the scRNA-Seq data (42). We found that most vectors of CD4^+^ T cells pointed toward SAT cells (Figure 9, C and D), suggesting that other CD4^+^ T cells might give rise to SAT cells.

**Figure 10:**
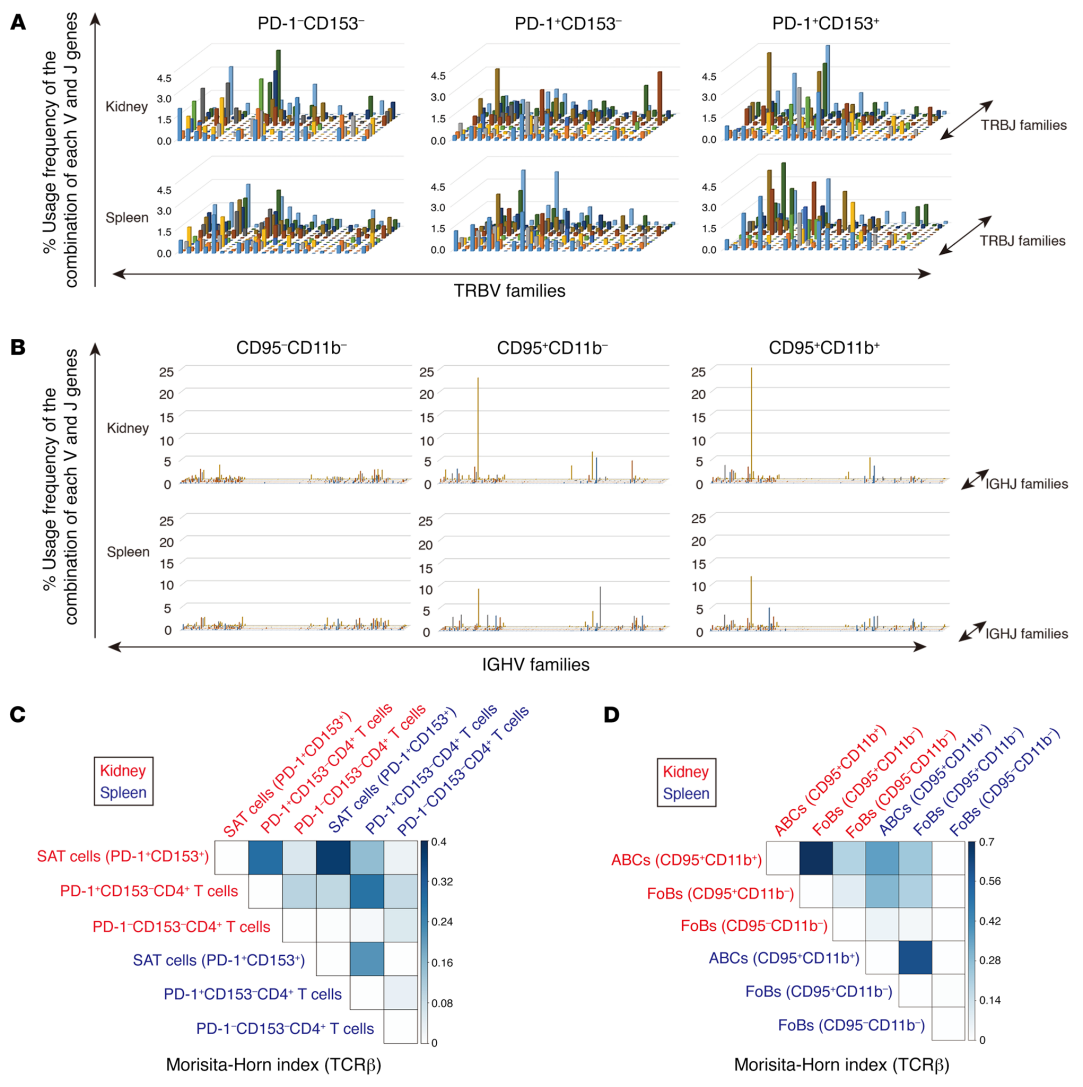
SAT cells and ABCs in aged injured kidney are derived from both local development and recruitment from the spleen. (**A**) T cell receptor β (TCRβ) repertoire clonalities of PD1^–^CD153^–^, PD1^+^CD153^–^, and PD1^+^CD153^+^CD44^hi^CD4^+^ T cells and (**B**) B cell receptor (BCR) repertoire clonalities of CD95^–^CD11b^–^, CD95^+^CD11b^–^, and CD95^+^CD11b^+^ B cells in aged injured kidneys and the spleen 45 days after ischemic reperfusion injury (IRI) induction. The *x* and *y* axes indicate the combination of V and J genes (TCR β chain V [TRBV] and TRBJ families; immunoglobulin heavy chain V [IGHV] and IGHJ families, respectively), and the *z* axis indicates the frequencies of usage. (**C** and **D**) Heatmap of similarity index (Morisita-Horn index) of (**C**) TCRβ clonotypes of 3 CD4^+^ T cell populations and (**D**) IgM BCR clonotypes of 3 B cell populations in aged injured kidneys and the spleen 45 days after IRI induction.

Next, we performed TCR and B cell receptor (BCR) repertoire analyses of SAT cells and ABCs, respectively, from aged injured kidneys and spleen, and compared them with those in other CD4^+^ T cells and B cells, respectively. All samples contained TCR and BCR clonotypes (Figure 10, A and B, and Supplemental Figure 8A), and several TCR and BCR clonotypes were shared between 3 CD4^+^ T cell populations and B cell populations in aged injured kidneys and spleen, respectively (Supplemental Figure 9). The diversity indices were similar between the subpopulations in both T and B cells (Supplemental Figure 8, B and C). TCRs of renal SAT cells exhibited intensive overlap with those of spleen SAT cells as well as renal PD-1^+^CD153^–^CD4^+^ T cells (Figure 10C and Supplemental Figure 10). Similarly, IgM BCRs of renal ABCs also exhibited intensive overlap with those of spleen ABCs as well as renal CD95^+^CD11b^–^ FoBs (Figure 10D). These results indicate that SAT cells and ABCs in the kidneys could be derived from both local differentiation from other CD4^+^ T cell populations and recruitment from the spleen.

### Human Tph/Tfh cells and ABCs in chronically inflamed tissues express TNFSF8 and TNFRSF8.

**Figure 11:**
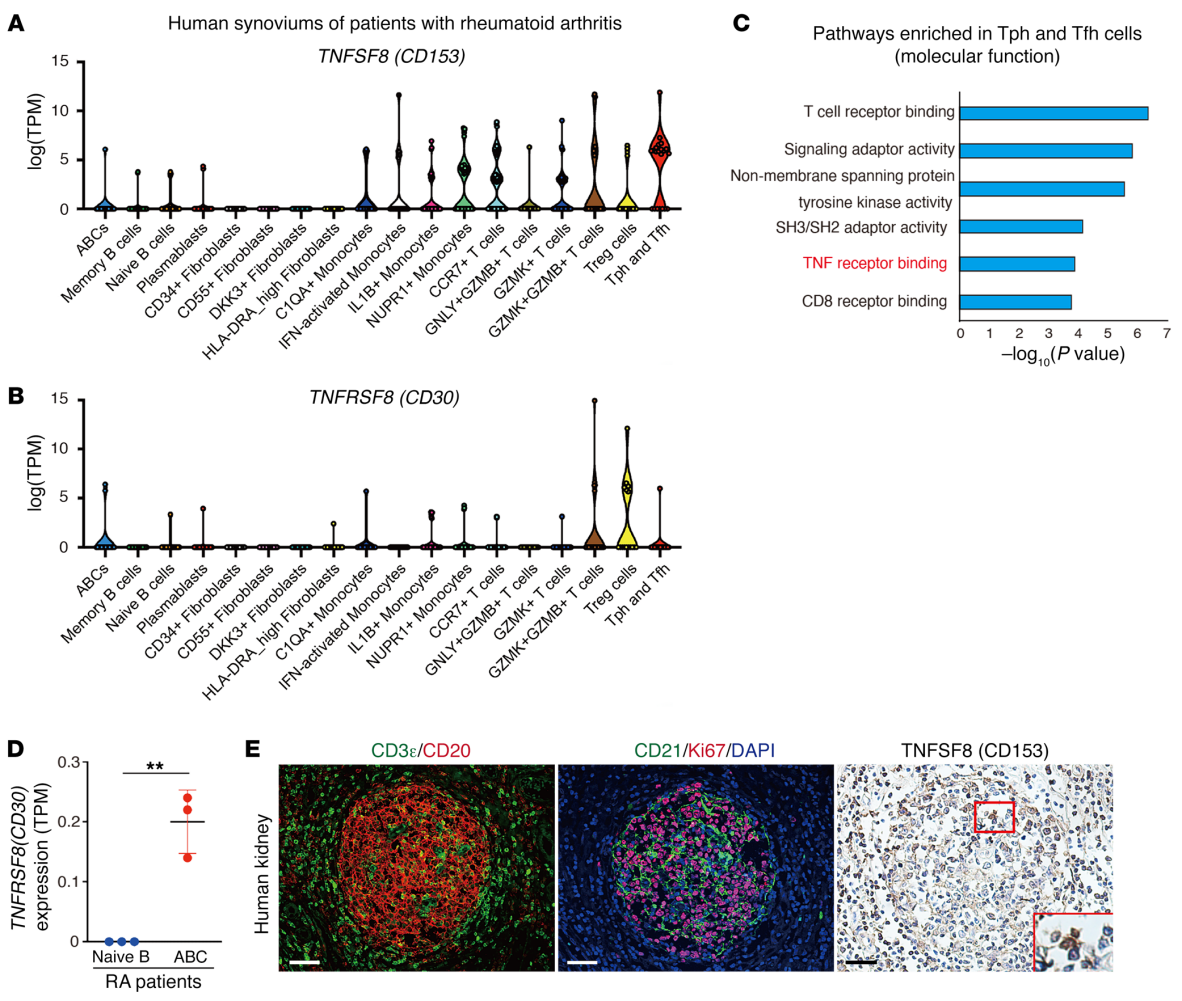
Human Tph/Tfh cells and ABCs express *TNFSF8* and *TNFRSF8*, respectively. (**A** and **B**) Reanalysis of published scRNA-Seq data of human samples. *TNFSF8* and *TNFRSF8* expression in various types of T cells and B cells in the synovium of patients with rheumatoid arthritis (RA) (log[TPM]). Data are shown as median (Kruskal-Wallis test followed by 2-sided Wilcoxon’s rank-sum test). (**C**) Enrichment analysis of molecular function ontology in Tph and Tfh cells in **A**. The *x* axis denotes the –log_10_ of enrichment *P* values calculated utilizing a *t* test for the enrichment of a specific pathway. (**D**) TPM values of *TNFRSF8* in naive B cells and ABCs from the peripheral blood of patients with RA (*n* = 3 per group). Data are presented as mean ± SD, and statistical significance was determined by an unpaired, 2-tailed *t* test. (**E**) Immunofluorescence of CD3ε and CD20, CD21 and Ki67, and immunohistochemistry of TNFSF8 in human kidneys. Magnified views of the outlined box are shown in the lower right. Scale bars: 50 μm. Data in **A**–**C** and **D** were derived from Zhang et al.’s (43) and Wang et al.’s (28) data sets, respectively. **P < 0.01.

Finally, we examined the expression levels of *TNFSF8* and *TNFRSF8* in aged human cells and tissues. First, we utilized a publicly available scRNA-Seq data set and examined the expression of *TNFSF8* and *TNFRSF8* in immune cells residing in synovial tissues from joints of aged patients with rheumatoid arthritis (RA) (43). Expression of *TNFSF8* and *TNFRSF8* was detected in more immune cell types compared with mice but was detected in CD4^+^ T cells with B cell helper functions (termed Tph or Tfh cells) and ABCs (Figure 11, A and B, and ref. 43). Additionally, gene ontology analyses of Tph/Tfh cells indicated that TNF receptor binding was included in the top 6 molecular functions (Figure 11C). We also examined *TNFRSF8* expression of B cells with bulk RNA-Seq data of peripheral blood cells of patients with RA (28) and confirmed the higher *TNFRSF8* expression in ABCs compared with naive B cells (Figure 11D). Immunohistochemical analysis utilizing serial sections of aged human kidney samples revealed TNFSF8-positive cells within TLTs, especially within the GCs (Figure 11E).

## Discussion

**Figure 12:**
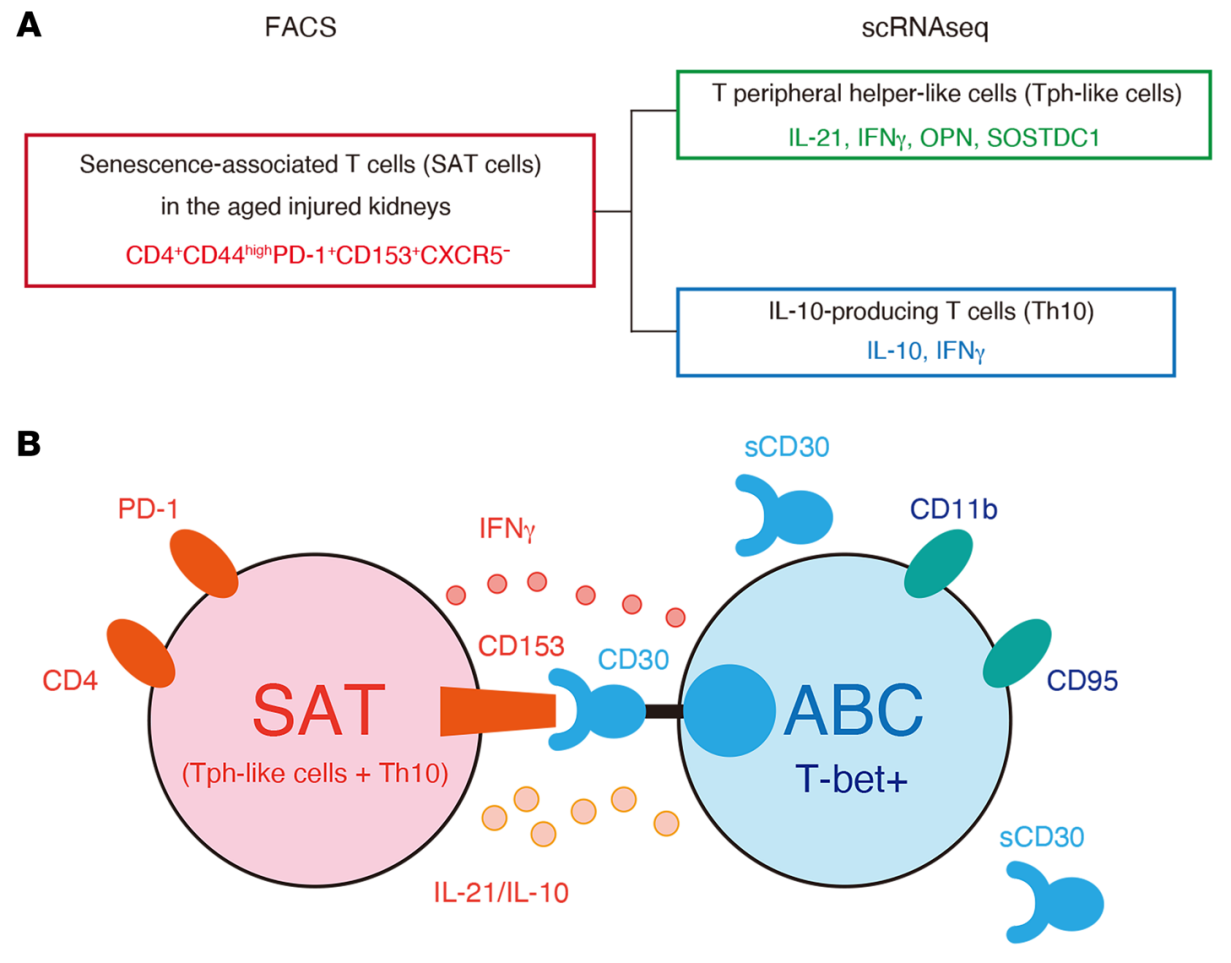
A model for interaction between SAT cells and ABCs within TLTs in aged injured kidneys. (**A**) SAT cells in aged injured kidneys are defined as CD153^+^CD44^hi^PD1^+^CXCR5^–^CD4^+^ T cells by flow cytometry. SAT cells are classified into Tph-like cells and Th10 at transcriptional levels. (**B**) SAT cells express PD-1 and CD153 on their surface, whereas ABCs are positive for transcriptional factor T-bet, and express CD11b and CD95 on their surface. SAT cells produce IL-21, IL-10, and IFN-γ, all of which are essential for the induction of ABCs and germinal center B (GCB) cells and the acquisition of these B cell helper functions of SAT cells is dependent on CD153/CD30 signaling. In the absence of CD153/CD30 signaling, SAT cells lose their B cell helper function and are not fully capable of inducing ABCs and GCB cells, resulting in a reduction in number and size of TLTs. Cell-surface CD30 is quickly lost after activation by shedding and becomes the soluble form of CD30 (sCD30).

Here, we show that CD153/CD30 signaling between 2 unique age-dependent lymphocyte subpopulations, SAT cells and ABCs, is required to promote age-dependent TLT expansion (Figure 12). SAT cells and ABCs gradually accumulated and coexisted within TLTs after injury. Genetic deletion of either CD153 or CD30 impaired functional SAT cell development and led to attenuated ABC and GCB cell development, resulting in a reduction in TLT number, size, and maturity, together with attenuated inflammation, fibrosis, and renal dysfunction. These data identify a nonredundant role for CD153/CD30 signalling in age-dependent TLT formation in aged injured kidneys, highlighting a previously unappreciated pathway with potential therapeutic targets for the treatment of kidney disease in the elderly.

The TNFSF of ligands and receptors is implicated in lymphocyte homeostasis and activation, and dysregulated signaling of these molecules can cause pathological conditions (44, 45). For example, while loss-of-function mutations in CD154 (*TNFSF5*) and CD40 (*TNFRSF5*) are linked to immunodeficiencies in mice and humans, gain-of-function alterations are associated with autoimmunity and B cell malignancy (44). However, most of the precise roles of both ligands and receptors of the TNFSF, especially in vivo, remain unknown (45). One of the reasons for this is that both ligands and receptors of the TNFSF can be expressed by various types of cells. Additionally, most TNFSF/TNFRSF members are not constitutively expressed, and their gene expression regulation is also variably dependent on the cell population, anatomical location, and the stage of differentiation of the cells expressing them (35, 45, 46). In the present study, utilizing scRNA-Seq combined with reporter mice analysis and ISH, we found that CD153/CD30 signaling is specific to SAT cells and ABCs in aged injured kidneys, respectively, and is essential for TLT formation. Bone marrow transplantation utilizing *Rag2^–/–^* mice further revealed that CD153 deficiency in immune cells, rather than resident cells, is essential for TLT formation and expansion. Notably, while genetic deficiency of CD153 or CD30 did not alter the expression levels of *Tbx21* and its downstream *Zeb2* as well as functional molecules in ABCs, the deficiency decreased the expression of genes related to ABC induction in SAT cells. These results indicate that CD153/CD30 signaling is indispensable for SAT cells to acquire B cell helper functions and the reduction of ABCs and GCB cells in *CD153^–/–^* and *CD30^–/–^* mice is secondary to the functional loss of SAT cells.

More importantly, we also demonstrated that *CD153^–/–^* mice were protected from kidney injury and exhibited preserved renal function. This suggests the therapeutic potential of targeting this signaling pathway to prevent kidney disease progression. Given that SAT cells and ABCs are induced under conditions that accelerate immune aging, such as autoimmune diseases (16, 23) and obesity (24, 25), the results in our study might be applicable to these pathological conditions. One potential caveat is that CD153/CD30 signaling also has host-protective roles to combat some infections. Although *CD153^–/–^* and *CD30^–/–^* mice have relatively minor defects in the ability to control nontuberculous mycobacteria (47), they have major defects in the control of infections with intracellular *Mycobacterium tuberculosis* (36). In particular, CD153 expression on CD4^+^ T cells is integral in combatting *M*. *tuberculosis* (36). Similarly, loss-of-function studies have demonstrated that T-bet^+^ B cells are required for optimal immune responses to various kinds of intracellular pathogens (48–50). Based on these dichotomous roles of the CD153/CD30 signaling pathway, the potential optimal approach for therapeutically targeting the CD153/CD30 pathway still needs to be defined in a context-dependent manner. Although CD153/CD30 signaling was found to be virtually specific to SAT cells and ABCs in this study, this signaling might not be specific to these cells in non-aging conditions. Further studies will also be required to clarify the role of CD153/CD30 signaling in non-aging pathological conditions.

One of the important requirements to better understand SAT cells and ABCs is to elucidate their origins. Based on the spatiotemporal association between SAT cell and ABC development, we considered the possibility that SAT cells and ABCs interact and develop together within TLTs from other CD4^+^ T cells and B cells, respectively, in aged injured kidneys. Indeed, we showed an in vivo developmental link between SAT cells and PD-1^+^CD153^–^CD4^+^ T cells, as well as ABCs and FoBs by TCR and BCR clonal sharing, in the kidney and spleen. Consistently, ABCs have been shown to be derived from FoBs, which is the major B cell population within TLTs (Figure 3E), by adoptive transfer studies and in vitro assays (21, 51). Additionally, molecular requirements for ABC development, such as IL-21 and IFN-γ, have also been determined (19, 51). By contrast, the cellular origin and initial molecular driver of SAT cells remain unexplored. In the present study, we provide an in vivo developmental link between SAT cells and PD-1^+^CD153^–^CD4^+^ T cells in the aged injured kidney as well as in the spleen by clone sharing. Further studies will be required to elucidate the molecular requirement and mechanism for SAT cell development. Additionally, shared TCR and BCR sequences were confirmed between SAT cells and ABCs in the kidney and spleen, respectively, suggesting the possible circulation of SAT cells and ABCs between the spleen and kidney. Understanding the relative contribution of local development and recruitment from the spleen during TLT formation is also important since it might provide insights into the more precise developmental mechanisms of TLTs, which requires further studies.

The crucial question is whether these findings could apply to TLT-associated pathological conditions in humans. In the present study, we confirmed the presence of *TNFSF8*-positive cells within TLTs in human kidneys, and *TNFSF8* and *TNFRSF8* expression in human Tph cells/Tfh cells and ABCs, respectively (Figure 11, A and B). Importantly, all synovial samples that contained Tph cells also had ABCs, but other patients lacked both types of cells (43), implying that Tph cells and ABCs are present in the same chronic inflammatory tissues. Additionally, in patients with systemic lupus erythematosus (SLE), a significant positive correlation between the frequency of Tph cells and both clinical disease activity and the frequency of CD11c^+^ B cells, which are recognized as ABC-like cells, has also been reported in peripheral blood (39). Consistently, the levels of sCD30 in peripheral blood were also increased in patients with RA and SLE (38), suggesting the activation of CD153/CD30 signaling in these diseases. Given that SAT cells and ABCs also contribute to SLE pathophysiology in murine models (16, 23), these results raise the hypothesis that the aberrant interactions between Tph and Tfh cells, which correspond to SAT cells in humans, and ABCs within TLTs might be a common pathological mechanism for driving chronic inflammation across multiple diseases and conserved across species. Actually, gluten-specific CD4^+^ T cells in patients with celiac disease have a phenotype equivalent to Tph cells and the same phenotypic cells were also detected in the patients with SLE and systemic sclerosis (52), suggesting that Tph cells represent a common driver of pathogenic B cell activation in various autoimmune diseases. One potential caveat is that, unlike in mice, the expression of *TNFSF8* and *TNFRSF8* mRNA was detected in broader types of immune cells in humans (Figure 11, A and B). Further studies are necessary to determine the cell types expressing CD153 on their cell surface and whether our current findings extend to immune aging-related pathological conditions in humans.

Our findings raise several important points that should be addressed in the future. First, further characterization of the CD153/CD30 pathway is needed. For example, in the absence of CD30, SAT cells lost B cell helper function but instead acquired proinflammatory characteristics, including IL-17–producing ability. This suggests that CD153/CD30 signaling is involved in CD4^+^ T cell differentiation and SAT cell development. Given that TNFSF members and their receptors can generate bidirectional signals and influence the survival, activation, or death of immune cells (46), CD153 reverse signals via CD30 from ABCs might play crucial roles in maintaining SAT cell functions. Further investigation is needed to clarify this possibility. Second, the precise relationships between SAT cells and other CD4^+^ T cell populations in aged injured kidneys with TLTs should be explored. A recent study has identified 3 age-dependent unique CD4^+^ T cell subsets in the spleen, including cytotoxic, exhausted (similar gene expression to SAT cells), and Treg (53). All of these also co-emerged in aged injured kidneys with multiple TLTs (Figure 3, A–C), suggesting a developmental link among these age-associated CD4^+^ T cells. Notably, the frequency of these subsets was closely associated with increased levels of proinflammatory cytokines in the sera (53). Given that age-dependent TLT formation occurs in various organs, it is intriguing to speculate that TLTs generate these age-associated T cells, together with ABCs, and promote inflammaging. Third, there is a possibility that CD153 and CD30 also have additional interacting molecules, since the effects of CD153 deficiency are more profound than those of CD30 deficiency (Figures 6 and 7). Further studies will be required to clarify the details of the CD153/CD30 signaling pathway.

In summary, our study exploring the cellular and molecular details in age-dependent TLT formation in the kidney have uncovered several findings relevant to immune aging and kidney disease. We found that 2 age-dependent lymphocytes, SAT cells and ABCs, accumulate within TLTs in the kidneys after injury, where they interact with one another via CD153 and CD30. This CD153-CD30 interaction is required for SAT cells to acquire ABC-inducing ability and essential for TLT formation. We additionally determined the therapeutic potential of CD153/CD30 signaling in kidney disease progression. Finally, we confirmed CD153 and CD30 expression in human Tph/Tfh cells and ABCs, respectively. Taken together, our findings elucidate a previously unappreciated role of CD153/CD30 signaling in age-dependent TLT formation and might guide the development of novel therapeutic strategies for kidney diseases in the elderly.

## Methods

### Animals.

We selected 8-week-old and 10- to 13-month-old male mice as the young and aged models, respectively. *Spp1*-EGFP-KI mice, *CD153^–/–^* mice, *Rag2^V^* mice, and *CD30^–/–^* mice were described previously (16, 47, 54–56). For other experiments, we purchased 8-week- and 12-month-old male C57BL/6J mice from Japan SLC as young and aged mice, respectively. Serum creatinine was measured by the creatininase–*N*-(3-sulfopropyl)-3-methoxy-5-methylaniline (creatinase-HMMPS) method (SRL Diagnostics). All mice were maintained in specific pathogen–free conditions in the animal facility of Kyoto University.

### Kidney injury models.

IRI and adenine nephropathy were induced as described previously (13). Briefly, IRI was induced by clamping the unilateral renal pedicles for 45 minutes. Adenine nephropathy was induced by feeding a 0.20 % adenine diet (Research Diets).

### Mononuclear cell isolation from the kidney, spleen, and blood.

After the collection of 500 μL of blood, mice were perfused with phosphate-buffered saline (PBS) and then the kidneys and spleens were isolated. The kidneys were minced finely and incubated in a water bath with collagenase/DNase I solution (1 mg/mL collagenase and 0.1 mg/mL DNase I in HBSS) for 20 minutes at 37°C, with gentle stirring. Digested kidneys were then centrifuged at 500*g* for 7 minutes. The resulting pellets were washed with fluorescence-activated cell sorting (FACS) buffer (2% FCS, 1% EDTA), and a single-cell suspension of the kidney digestion was obtained by filtering through a 70-μm cell strainer. A single-cell suspension of splenocytes was generated by the mechanical disruption of tissue. Red blood cells were lysed with RBC lysis buffer (Invitrogen) for 5 minutes.

### Flow cytometric analysis and cell sorting.

Single-cell suspensions were blocked with FcR Blocking reagent (Miltenyi Biotec) at 4°C for 20 minutes, and then cells were stained with cocktails of conjugated antibodies against the following proteins: mouse CD45 (BioLegend, clone 30-F11, catalog number 103112), CD3ε (BioLegend, clone 1452C-11 conjugated with FITC, catalog number 100306), CD4 (eBioscience, clone GK1.5 conjugated with PE-Cy7, catalog number 60-0041-U100), CD8a (BioLegend, clone 53-6.7 conjugated with Pacific Blue, catalog number 100725), B220 (eBioscience, clone RA3-6B2 conjugated with APC–eFluor 488, catalog number 47-0452-82), CD44 (Thermo Fisher Scientific, clone IM7 conjugated with APC–eFluor 488, catalog number 47-0441-82), CD11b (eBioscience, clone M1/70 conjugated with violetFluor 450, catalog number 75-0112-U100), CD11c (BioLegend, clone N418 conjugated with PE-Cy7, catalog number 117317), PD-1 (eBioscience, clone J43 conjugated with eFluor 450, catalog number 48-9985-82), CD153 (eBioscience, clone RM153 conjugated with PE, catalog number 12-1531-83), CD30 (Biolegend, clone mCD30.1 conjugated with PE, catalog number 10236), biotin-CXCR5 (BD Pharmingen, clone 2G8, catalog number 551960), APC-Streptavidin (BioLegend, catalog number 405207), CD95 (BD Pharmingen, clone Jo2 conjugated with PE-Cy7, catalog number 557653), GL7 (BD Pharmingen, clone GL7 [RUO] conjugated with FITC, catalog number 553666), IFN-γ (BioLegend, clone XMG1.2 conjugated with FITC, catalog number 505806), IL-17A (BioLegend, clone TC11-18H10.1 conjugated with APC, catalog number 506915), IL-4 (BioLegend, clone 11B11 conjugated with Alexa Fluor 488, catalog number 504109), and OPN (R&D Systems, PE conjugated, catalog number IC808P) at 4°C for 20 minutes. For intracellular T-bet staining, cells were surface stained, washed in PBS, permeabilized with Foxp3/Transcription Factor Staining buffer set (eBioscience, catalog number 00-5523-00), and stained with anti–mouse T-bet (eBioscience, clone 4B10 conjugated with PE, catalog number 12-5825-80). For intracellular cytokine staining, cells were cultured with PMA (20 ng/mL) and ionomycin (1 μg/mL) in the presence of brefeldin A for 6 hours before analysis. Flow cytometric analyses and cell sorting were performed using a FACSCanto and FACSAria II/III (BD Biosciences) and analyzed with FlowJo software (Becton Dickinson Co.). An example of our generalized gating strategy for SAT cells can be seen in Supplemental Figure 11.

### Bulk RNA-Seq library preparation and sequencing.

SAT cells, other CD4^+^ T cell populations, and ABCs in aged injured kidneys 45 days after IRI induction were sorted using a FACSAria II/III. In aged WT mice, each CD4^+^ T cell subpopulation and ABCs were isolated from the pooled cells of 2 individual aged mouse kidneys subjected to IRI (Figure 1G, Figure 6H, Figure 7H, and Figure 7I; *n* = 3 from independent experiments). In contrast, in aged *CD30^–/–^* mice, SAT cells were isolated from the pooled cells of 3 individual aged mouse kidneys subjected to IRI per sample (Figure 7H; *n* = 3 from independent experiments). In aged *CD153^–/–^* and *CD30^–/–^* mice, ABCs were isolated from the pooled cells of 3 individual aged mouse kidneys subjected to IRI per sample (Figure 6H and Figure 7I; *n* = 3 from independent experiments). RNA was harvested using TRIzol reagent (Thermo Fisher Scientific). Library preparation, sequencing, and data analysis were performed by DNAFORM. Quality and quantity of extracted RNA were assessed by NanoDrop 8000 Microvolume UV-Vis spectrophotometer (Thermo Fisher Scientific) and Agilent RNA 6000 Nano kit of the BioAnalyzer 2100 System (Agilent Technologies). The RNA-Seq library was prepared using a SMARTer Stranded Total RNA-Seq Kit v2 - Pico Input Mammalian (Takara Bio) following the manufacturer’s instructions. In brief, first-strand cDNA synthesis was performed using N6 primer. A first round of PCR amplification added full-length Illumina adapters, including barcodes. After that, the cDNA derived from rRNA was depleted by ZapR. Remaining library fragments originating from non-rRNA were amplified using universal primers. The libraries were sequenced on a HiSeq sequencer (Illumina) to generate 150-nt paired-end reads. The RNA-Seq data presented in this study have been deposited at the DNA Bank of Japan (DDBJ; http://www.ddbj.nig.ac.jp/index-e.html) under accession number DRA011230.

### Bulk RNA-Seq data analysis.

For all of our data sets, reads were trimmed to remove barcode sequences using Trimmomatic version 0.36 (57). The resultant reads were mapped to mouse (mm10; STAR version 2.6.0c with Gencode vM25 comprehensive set; https://github.com/alexdobin/STAR) reference gene models. Indexing was performed with the following parameters: --runThreadN 10 --runMode genomeGenerate --genomeFastaFiles genome.fa --sjdbGTFfile Gencodeannotation.gtf --sjdbOverhang 100. Mapping was performed with the following parameters: --runThreadN 20 --outSAMtype BAM SortedByCoordinate --quantMode TranscriptomeSAM --outFilterMultimapNmax 1 --readFilesCommand zcat. The expected counts as output from RSEM were then normalized on the basis of a trimmed mean of M values (TMM) and differential expression analyses were conducted using edgeR version 3.30.3 (https://bioconductor.org/packages/release/bioc/html/edgeR.html). Gene ontology analysis was performed using Gene Set Enrichment Analysis (GSEA) v3 (http://www.gsea-msigdb.org/gsea/index.jsp). Heatmap and scatter plots were generated utilizing pheatmap v1.0.12 (https://cran.r-project.org/web/packages/pheatmap/index.html) and ggpubr v0.4.0 (https://cran.r-project.org/web/packages/ggpubr/index.html), respectively.

### scRNA-Seq.

Two aged male C57BL/6J mice were used. FACS-isolated CD45^+^ cells from aged injured kidneys 45 days after IRI induction were loaded onto a Chromium Single Cell Controller using the Chromium Single Cell 3′ Library and Gel Bead Kit v3 (both from 10× Genomics) following the manufacturer’s instructions. Sample processing and library preparation were performed according to the manufacturer’s instructions. The libraries were sequenced on a HiSeq sequencer (Illumina). The scRNA-Seq data in the present study have also been deposited in the DDBJ (http://www.ddbj.nig.ac.jp/index-e.html) under accession number DRA011230.

### scRNA-Seq data processing.

Processing of sequencing data was performed with the Cell Ranger pipeline version 3.0.2 (10× Genomics) with mm10 v.3.0.0 Cell Ranger reference. The subsequent data analysis was performed using R package Seurat v3.1.5. We followed the Seurat vignette (https://satijalab.org/seurat/pbmc3k_tutorial.html) to create the Seurat data matrix object. In brief, we kept all genes expressed in more than 3 cells and cells with at least 200 detected genes. Cells with mitochondrial gene percentages greater than 10% and unique gene counts greater than 4500 or less than 200 were discarded. The data were normalized using Seurat’s “NormalizeData” function, in which unique molecular identifier (UMI) counts for each gene from each cell were divided by the total UMI counts from that cell, multiplied by a scale factor of 10,000, and natural log transformed. Highly variable genes were then identified using the function “FindVariableGenes” in Seurat with default parameters. We also regressed out the variation arising from library size and percentage of mitochondrial genes using the function “ScaleData” in Seurat. Principal component analysis (PCA) dimensionality reduction was performed using the Seurat “Run-PCA” function. The computed PCs were used to perform unsupervised cell clustering using the function “FindClusters” and to generate uniform manifold approximation and projection (UMAP) plots of scRNA-Seq data using the Seurat “RunUMAP” function. Cluster marker genes were identified using the “FindAllMarkers” function with the following settings: min pct = 0.25, logfc.threshold = 0.25. For enrichment analysis of biological process ontology, cluster marker genes were analyzed in DAVID (ref. 58 and Supplemental Figure 2B).

### Cell clustering.

Clustering of CD45^+^ cells in the kidneys was performed by Seurat in a stepwise manner. We initially performed low-resolution clustering, analyzing all CD45^+^ cells together, and then annotated each of the resulting clusters as CD4^+^ T cells, CD8^+^ T cells, B cells, plasmablasts, dendritic cells, macrophages, double-negative T cells plus macrophages, proliferating cells, and neutrophils, as described in the main text. The Seurat “FindAllMarkers” function was used to find markers for each of the identified clusters. The CD4^+^ T cells and B cells were then integrated for further subclustering. All details regarding the Seurat analysis performed in the present study can be found on the website tutorial (http://satijalab.org/seurat/v3.0/pbmc3k_tutorial.html).

### CellPhoneDB analysis and RNA velocity analysis.

CellPhoneDB v.2.0 was used for the receptor-ligand analysis; focused analysis of TNFSF members is shown in Figure 5A. RNA velocity (42) was used to assess the transcriptional dynamics of cells (Figure 9D).

### Statistics.

Statistical analysis was performed by Student’s *t* test, 1-way analysis of variance (ANOVA) with Bonferroni’s post hoc test, Pearson’s correlation analysis, a nonparametric test for trend (Cuzick’s test for trend), and the Kruskal–Wallis test followed by the 2-sided Wilcoxon’s rank-sum test as indicated in the figure legends, with significance set at *P* less than 0.05. Statistical analysis was performed using JMP software (v14, SAS Institute Inc.) and R v4.0.2.

### Study approval.

All animal experiments were approved by the Animal Research Committee, Graduate School of Medicine, Kyoto University (MedKyoto20187), and were conducted in accordance with the *Guide for the Care and Use of Laboratory Animals* (National Academies Press, 2011). All human specimens were procured and analyzed after obtaining written informed consent and with approval of the ethics committees of RWTH University of Aachen Hospitals (EK206/09, EK244-14, and EK042-17) and Graduate School of Medicine, Kyoto University (R0254).

## Author contributions

Y Sato and MY designed the experiments and wrote the manuscript. Y Sato performed experiments and analyzed data. AO, NT, T Yoshikawa, XC, JS, T Yamanashi, T Yamamoto, and YM contributed to bioinformatic analysis. YF, HF, and KM contributed to cell sorting and FACS analysis. KT, MK, TH, SK, Y Shimizu, LJ, YK, and ST performed some experiments and collected data. PB and JF contributed to collecting human samples and data. YH, MH, YY, HK, YM, and NM discussed the results and commented on the manuscript.

## Supplementary Material

Supplemental data

Supplemental data set 1

## Version 1 11/23/2021

In-Press Preview

## Version 2 01/18/2022

Electronic publication
