# Supplementary material for: CD153/CD30 signaling promotes age-dependent tertiary lymphoid tissue expansion and kidney injury
Source: J Clin Invest. 2022 Jan 18;132(2):e146071. doi: 10.1172/JCI146071 (PMC8759786; doi:10.1172/JCI146071)
Supplement: Supplemental data [file jci-132-146071-s056.pdf]

Supplemental Materials for

CD153-CD30 signaling promotes age-dependent tertiary lymphoid tissue expansion and kidney injury

Yuki Sato, Akiko Oguchi, Yuji Fukushima, Kyoko Masuda, Naoya Toriu, Keisuke Taniguchi, Takahisa Yoshikawa, Xiaotong Cui, Makiko Kondo, Takeshi Hosoi, Shota Komidori, Yoko Shimizu, Harumi Fujita, Li Jiang, Yingyi Kong, Takashi Yamanashi, Jun Seita, Takuya Yamamoto, Shinya Toyokuni, Yoko Hamazaki, Masakazu Hattori, Yasunobu Yoshikai, Peter Boor, Jürgen Floege, Hiroshi Kawamoto, Yasuhiro Murakawa, Nagahiro Minato and Motoko Yanagita

Corresponding author:

Motoko Yanagita and Yuki Sato: Department of Nephrology, Graduate School of Medicine, Kyoto University, Shogoin-Kawahara-cho 54, Sakyo-ku, Kyoto 606-8507, Japan.

E-mail: motoy@kuhp.kyoto-u.ac.jp, yukisato@kuhp.kyoto-u.ac.jp,Tel: +81-75-751-3860, Fax: +81-75-751-3859

Supplemental Materials

Material and Methods

References for Supplemental materials

Supplemental Figure 1-11

Supplemental Table 1. Primer sequences used for real-time PCR.

Other Supplemental Materials for this manuscript includes the following:

Data file S1 (Microsoft Excel format). TCR and BCR sequences for $CD4^+$ T cell and B cell subpopulations in aged injured kidneys and spleens.

Material and Methods

Identification and quantification of TLTs

In the present study, we defined TLTs as organized lymphocyte clusters exhibiting the signs of proliferation, as previously described (1). Identification and diagnosis of TLTs in the renal cortex was based on the unique perivascular localization of mononuclear cell aggregates by light microscopy and the presence of lymphocyte aggregates with Ki67 signals inside by immunofluorescence. Determination of TLT stages in the renal cortex was examined with immunofluorescence of (i) CD3ε and CD20 and (ii) Ki67 and CD21 in two serial sections for each mouse and human, and was assessed by an experienced renal pathologist. TLTs containing FDC were defined as advanced-stage TLTs. The number of TLTs was shown as the number per one section.

Quantification of TLT size

The renal TLT sizes were examined in the PAS-stained sections of injured kidneys as described previously (2). TLT size was defined as the total cumulative size of the TLTs in the renal cortex of the sample. Pictures that included TLTs were taken with the same size and resolution, and TLT size was measured by an experienced renal pathologist using Adobe Photoshop software.

Renal histochemistry

Mouse kidneys were harvested, cut along the short axis at the maximum area of the whole kidney, fixed in Carnoy's solution, embedded in paraffin, sectioned (2.0 μm thickness), and then stained with periodic acid–Schiff (PAS) or Masson's trichrome (MTC).

Renal immunofluorescence

For immunofluorescence studies of mouse kidneys, kidneys were fixed in 4% paraformaldehyde

(PFA), incubated in 20% sucrose for 6 h, and then incubated in 30% sucrose in PBS at 4°C overnight. Optimal cutting temperature compound (OCT)-embedded (Sakura Finetek) kidneys were cryosectioned into 6.0 µm sections and mounted on Superfrost slides (Matsunami Glass). Sections were blocked with 5% serum appropriate to the secondary antibody for 1 h at room temperature, and then incubated overnight at 4°C with primary antibodies. The following primary antibodies were used in the mouse immunohistochemistry and immunofluorescence experiments: anti-GFP (catalog ab13970; abcam), -Ki67 (catalog 14-5698; eBioscience, clone SolA15), -CD45 (catalog 14-0451; eBioscience, clone 30-F11), -CD11c-PE (catalog 12-0114; eBioscience, clone N418), -CD21 (catalog ab75985; abcam, clone EP3093), -Calbindin-D28K (catalog C9848, Sigma-Aldrich, clone CB-955), -p21 (catalog ab188224; abcam, clone EPR18021), -p75NTR (catalog AF1157; R&D Systems), -CD3ε (catalog 550275; BD Pharmingen, clone 17A2), -B220 (catalog 557390; BD PharMingen, clone RA3-6B2), Tamm–Horsfall protein (catalog BT-590; Biomedical Technologies Inc), -NCC (catalog AB3553; Chemicon International) antibodies, and Alexa-conjugated phalloidin (catalog A22287; Thermo Fisher Scientific). EGFP was visualized with the anti-GFP antibody.

For immunofluorescence studies of human kidneys, kidney sections were fixed in formalin, embedded in paraffin, and sectioned (3.0-µm thickness). The paraffin-embedded sections were deparaffinized with xylene, rehydrated, and then steam-heated for 15 min. The sections were incubated with 5% serum appropriate to the secondary antibody for 1 h at room temperature, and then incubated overnight at 4°C with the following primary antibodies: anti-CD20 (catalog 14-0202; eBioscience, clone L26), -CD3ε (catalog ab5690; abcam), -Ki67 (catalog NCL-Ki67p; Vision BioSystems) and -CD21 (catalog ab75985; abcam, clone EP3093) antibodies.

All staining samples were visualized using the appropriate secondary antibodies (1/300), counterstained with DAPI, and analyzed using a confocal microscope (FV1000D; Olympus).

Renal immunohistochemistry

Human kidneys were fixed in formalin, embedded in paraffin, and sectioned (3.0 μm thickness). For immunohistochemistry, paraffin-embedded tissue sections were rehydrated, and endogenous peroxidase was blocked using 3% H_2O_2. Antigen retrieval was performed with trypsin antigen-retrieval solution (catalog ab970; Abcam), and then tissue sections were stained with anti-CD153 primary antibody (catalog PAB26949; Abnova). Antibody labeling was performed with a DAB reagent (Vector Laboratories). Sections were counterstained with hematoxylin.

RNA in situ hybridization (RNA-ISH) of Tnfsf8 and Tnfrsf8

Detection of mouse *Tnfsf8 and Tnfrsf8* RNA was performed on cryosections described above and FFPE sections of mouse kidneys subject to 45 min IRI at day 30 and 45 utilizing Advanced Cell Diagnostics (ACD) RNAscope 2.5 HD Reagent Kit-Brown (#322300; ACD) and RNAscope Target Probes Mm-Tnfsf8 (Lot: 18205A; ACD) and Mm-Tnfrsf8 (Lot: 18033c; ACD) according to the manufacturer's instructions.

Soluble CD30 concentration in plasma

The levels of sCD30 were measured in murine plasma specimens with an enzyme-linked immunosorbent assay (ELISA) kit (catalog DY852; R&D Systems) according to the manufacturer's instructions.

Bone marrow transplantation experiments

Bone marrow transplantation experiments were performed as previously described (3). Briefly, bone marrow cells obtained from donor mice were washed with cold PBS and injected intravenously (3.0×10^6 cells) into 4 Gy-irradiated 10-month-old male Rag2 knockout mice. After 6 weeks, the mice were subjected to unilateral renal IRI.

Quantitative analysis for renal fibrosis and tubular injury

Fibrosis score was quantified as αSMA-positive area (pixel) in the renal interstitial space as described previously (1). Briefly, six images of each kidney section at the cortical and cortico-medullary field, except for the TLT area, were taken. All images were obtained with the same laser power and gain intensity using a confocal microscope (FV1000D; Olympus). αSMA-positive areas, except for vascular smooth muscle cells, were automatically calculated by Adobe Photoshop software. Tubular injury scores were examined in PAS-stained sections of aged kidneys 28 days after the initiation of adenine feeding. Tubular injury scores were graded by an experienced renal pathologist blinded to the sample information using a semi-quantitative scale. In brief, five images of each kidney section in the cortical area, except for the TLT area, were taken. The extent of necrosis, cell loss, and tubular atrophy of the renal cortex was graded as follows: 0, normal kidney; 0.5, <10%; 1, 10–25%; 2, 25–50%; 3, 50–75%; and 4, 75–100%.

Real-time RT-PCR analysis

RNA extraction and real-time reverse transcription polymerase chain reaction (RT-PCR) were performed as described previously (2). The primer sequences are listed in Table S1. Expression levels were normalized to those of *Gapdh* and expressed relative to the levels in aged wild-type kidneys on day 0 (IRI) (Figure 1D), in aged CD153$^{-/-}$ and CD30$^{-/-}$ mouse kidneys subject to IRI on day 45 (Figure 6B and Figure 7B, respectively), and in aged CD153$^{-/-}$ mouse kidneys subject to adenine nephropathy on day 28 (Figure 7I).

TCR and BCR repertoire analysis

CD4^{+} T cell populations and B cell populations were isolated from single-cell suspensions of the spleens and kidneys of two 12-month-old male C57BL/6J mice 45 days after unilateral IRI inductions using FACS Aria II (BD Bioscience). Total RNA was extracted from CD4^{+} T cells and B cells using a RNeasy Mini Kit (Qiagen) following the manufacturer's recommended protocol. Next-generation sequencing was performed with unbiased T cell receptor (TCR)/ B cell

receptor (BCR) repertoire analysis technology (Repertoire Genesis Inc.). Unbiased adaptor-ligation PCR was performed as described previously (4, 5). In brief, total RNA was converted to cDNA with Superscript III reverse transcriptase (Thermo Fisher Scientific). Subsequently, double-strand cDNA (ds-cDNA) was synthesized, and an adaptor was ligated to the 5' end of the ds-cDNA and then cut with Sph I restriction enzyme. For TCR analysis, PCR was performed using P20EA adaptor primer and TCRα-chain constant region-specific (mCA1) or TCRαβ-chain constant region-specific (mCB1) primers. The second PCR was performed using P20EA and either mCA2 or mCB2 with the same PCR conditions. For BCR analysis, P20EA and IgM constant region-specific (mCM1) or IgG constant region-specific (mCG1) primers were used for the first PCR and then mCM2 or mCG2 primer was used for the second PCR. After Tag PCR amplification, index sequences were added by amplification with a Nextera XT index kit v2 setA (Illumina). Sequencing was performed with the Illumina Miseq paired-end platform (2 × 300 bp). Data processing, assignment, and data aggregation were performed with repertoire analysis software. TCR and BCR sequences were assigned with the International ImMunoGeneTics (IMGT) reference sequences (http://www.imgt.org). Nucleotide sequences of complementarity-determining region 3 (CDR3) ranged from conserved cysteine at position 104 (Cys104) of the IMGT nomenclature to conserved phenylalanine at position 118 (Phe118) and the following glycine (Gly119) was translated into amino acid sequences, and the identical V (TCR chain V (TRV) or immunoglobulin heavy chain V (IGHV)), J (TRJ or IGH), and deduced amino acid sequence of the CDR3 were defined as a unique sequence read (USR). The copy number of USRs was automatically counted by RG software. TCR and BCR diversity analysis was performed according to the method described previously (6, 7). The Morisita–Horn similarity index was calculated by 'vegan' package version 2.5-6, and the Venn diagram was depicted by VennDiagram package version 1.6.20 with R version 4.0.2. TCR and BCR sequences are listed in Data file S1.

Reanalysis of published human RNA-seq data

Normalized RNA-seq data available in the National Center for Biotechnology Information's Gene Expression Omnibus (accession number GSE110999) were used and further analyzed in R. Detailed methods of data normalization were described in previously published articles (8).

Reanalysis of published human scRNA-seq data

scRNA-seq data of joint synovial tissues from patients with RA (9), which is a data from the Accelerating Medicines Partnership project, were preprocessed by Cincinnati Children's Hospital Medical Center's ToppCell project, and a normalized data matrix in logTPM was obtained. Pooled samples from 18 rheumatoid arthritis patients (3 leukocyte poor RA: mean age 64.2 years old and 15 leukocyte-rich RA: mean age was 57.3 years old) and 3 osteoarthritis patients (mean age 71years old) were used in analysis (9).

Analysis of human kidney specimens

All human specimens were procured and analyzed after obtaining informed consent and with the approval of the ethics committee at RWTH University of Aachen Hospitals (Aachen, Germany) and Kyoto University Hospital (Kyoto, Japan). Kidneys from patients with chronic pyelonephritis who underwent nephrectomy at RWTH University of Aachen Hospitals were analyzed (1).

References

1. Sato Y, et al. Developmental stages of tertiary lymphoid tissue reflect local injury and inflammation in mouse and human kidneys. *Kidney Int.* 2020;98(2):448-63.
2. Sato Y, et al. Heterogeneous fibroblasts underlie age-dependent tertiary lymphoid tissues in the kidney. *JCI Insight.* 2016;1(11):e87680.
3. Ikawa T, et al. Induced Developmental Arrest of Early Hematopoietic Progenitors Leads to the Generation of Leukocyte Stem Cells. *Stem Cell Reports.* 2015;5(5):716-27.
4. Sato-Kaneko F, et al. Combination immunotherapy with TLR agonists and checkpoint inhibitors suppresses head and neck cancer. *JCI Insight.* 2017;2(18).
5. Shalapour S, et al. Inflammation-induced IgA^+ cells dismantle anti-liver cancer immunity. *Nature.* 2017;551(7680):340-5.
6. Ricotta C. Through the jungle of biological diversity. *Acta Biotheor.* 2005;53(1):29-38.
7. Rempala GA, and Seweryn M. Methods for diversity and overlap analysis in T-cell receptor populations. *J Math Biol.* 2013;67(6-7):1339-68.
8. Wang S, et al. IL-21 drives expansion and plasma cell differentiation of autoreactive $CD11c^{hi}T\text{-}bet^+$ B cells in SLE. *Nat Commun.* 2018;9(1):1758.
9. Zhang F, et al. Defining inflammatory cell states in rheumatoid arthritis joint synovial tissues by integrating single-cell transcriptomics and mass cytometry. *Nat Immunol.* 2019;20(7):928-42.

Supplemental Figure 1. Histological analysis of wild-type aged kidneys subject to ischemic reperfusion injury (IRI).

Representative images of aged kidneys 4, 24, 60 days after 45min IRI by PAS staining. Yellow arrow heads indicate the localization of tertiary lymphoid tissues (TLTs). Scale bars: 300 μm.

Supplemental Figure 2. Immune cell profiling in aged injured kidneys.

(A) Violin plots showing the expression of canonical marker genes across all 14 subsets of $CD45^+$ cells. Fourteen clusters of $CD45^+$ cells were identified (C0-C13) and their putative identities were specified in the box. **(B)** Gene ontology enrichment analysis of upregulated genes in cluster 12. The x axis denotes the -log10 of enrichment P values by modified Fisher's exact test. **(C)** Violin plots showing the expressions of *Cd3e*, *Cd4*, *Ccr7*, *Angptl2*, *Il2ra*, *Gzmk*, *Cxcr6*, and *Lag3* in six clusters of $CD4^+$ T cells in aged injured kidneys 45 days after IRI. The putative identities of each cluster were specified in the box. **(D)** IFNγ, IL-4 and IL-17 production by $CD4^+$ T cells from aged injured kidneys, represented as the percentage of positive cells (n = 4/group). Cells were stimulated in bulk culture with PMA and ionomycin. Values are shown as means ± SD, and statistical significance was determined by one-way ANOVA with Bonferroni post hoc analysis. ***$P < 0.001$.

Supplemental Figure 3. *Spp1* are expressed in part of the distal tubules in the kidneys under physiological condition.

Immunofluorescence of GFP and phalloidin (a marker for proximal tubules), Tamm-Horsfall protein (THP) (a marker for thick ascending limbs) and thiazide-sensitive sodium chloride co-transporter (NCC) (a marker for distal convoluted tubules), and calbindin D28K (a marker for distal convoluted tubules) in the kidneys of aged *Spp1*-EGFP-knockin mice under physiological condition. EGFP was visualized with an anti-GFP antibody. Scale bar: 50 μm.

Supplemental Figure 4. **Both CD21$^+$ and CD21$^-$ B cells exist within TLTs.**

Immunofluorescence of CD21 and B220 in aged injured kidneys 45 days after IRI induction. A magnified view of the outlined box on the left is shown on the right. Scale bars: 50 μm. WT: wild type.

Supplemental Figure 5. ***Tnfsf8* and *Tnfrsf8* mRNA expression are almost confined within TLTs in aged injured kidneys.** In situ hybridization of *Tnfsf8* (gene name of CD153) and *Tnfrsf8* (gene name of CD30) in aged kidneys 24 days after IRI induction. The red dotted lines indicate the localization of TLTs. Scale bars: 100μm.

Supplemental Figure 6. *Tnfrsf8* mRNA expression are almost undetectable before TLT development in aged injured kidneys. (A) Expression levels of *Tnfsf8* (gene name of CD30) mRNA (n = 4/time point) in aged mouse kidneys following IRI. **(B)** Representative in situ hybridization of *Tnfrsf8* with immunofluorescent co-staining for αSMA in aged kidneys 4 days after IRI induction. Scale bar: 50μm.

Supplemental Figure 7. **Aged *CD153$^{-/-}$* and *CD30$^{-/-}$* mice exhibit normal renal function and structure under physiological condition. (A-C)** Histology (PAS staining), **(D)** total glomeruli number per section and **(E)** renal function in aged wild-type (WT), CD153-deficient (*CD153$^{-/-}$*) and CD30-deficient (*CD30$^{-/-}$*) mice under physiological condition (n = 4/group). Values are shown as means ± SD, and statistical significance was determined by one-way ANOVA. Scale bars: upper panels 300 μm, lower panels: 50 μm.

Supplemental Figure 8. **Repertoire analysis of CD4$^+$ T cells and B cells in aged injured kidneys and spleens.**

(A) TCRα repertoire clonalities of three CD4$^+$ T cell subpopulations in aged injured kidneys and spleen. The *x* and *y* axes indicate the combination of V and J genes (TCR α chain V (TRAV) and TRAJ families), and the *z* axis indicates their frequencies of usage. **(B-C)** Diversity index (Shannon-Weaver index) of **(B)** TCRα and TCRβ of three CD4$^+$ T cell subpopulations and **(C)** IgM B cell receptor (BCR) of three B cell subpopulations in aged kidneys and spleens 45 days after IRI induction.

Supplemental Figure 9. **Absolute numbers of T cell receptor (TCR) and B cell receptor (BCR) clone in aged injured kidneys and spleens. (A-B)** Venn diagram depicting the absolute number of TCRα and TCRβ clonotype shared across **(A)** three CD4$^+$ T cell populations and shared across **(B)** PD1$^+$CD153$^-$ and PD1$^+$CD153$^+$ CD4$^+$ T cell populations in aged kidneys and the spleen 45 days after IRI induction. **(C-D)** Venn diagram depicting the absolute number of IgM BCR clonotype shared across three B cell subpopulations within the same organs and across **(D)** CD95$^+$CD11b$^+$ and CD95$^+$CD11b$^-$ B cell populations in aged kidneys and the spleens 45 days after IRI induction.

Supplemental Figure 10. **Clone sharing between SAT cells and other CD4$^+$ T cells in aged injured kidney and spleen.** Heatmap of similarity index (Morishita-Horn index) of TCRα clonotype of three CD4$^+$ T cell populations in aged injured kidneys and the spleen 45 days after IRI induction.

Supplemental Figure 11. Gating strategy to define senescence-associated T (SAT) cells. Representative gating strategy of SAT cells from aged injured kidneys 45 days after IRI induction. Lymphocytes were gated according to their FSC-A/SSC-A profile to exclude debris, and then doublets were also excluded by the FSC-W/FSC-H profile. Subsequently, single cells were gated

on live cells and then gated on CD45^{+} cells. To define SAT cells, CD45^{+} cells were gated on CD3ε^{+}CD44^{high}, followed by CD4^{+} populations, and then PD1^{+}CD153^{+} T cells were plotted.

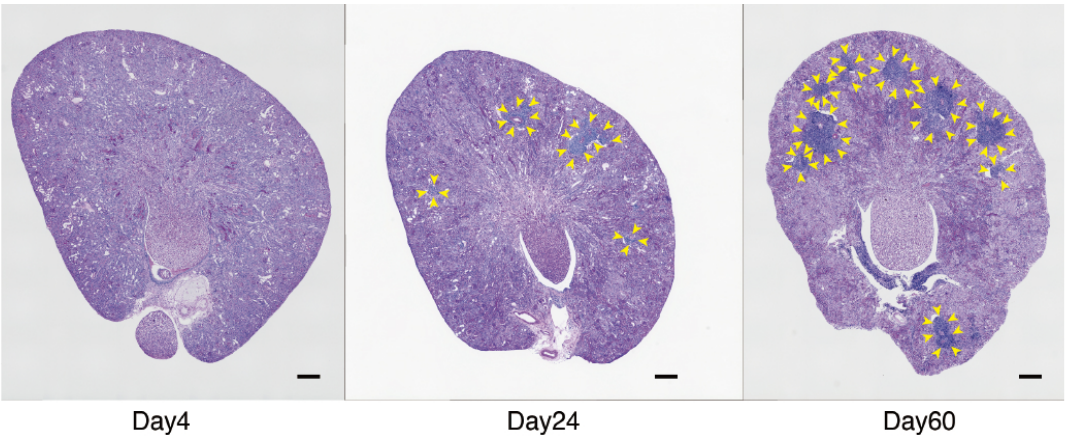

Supplemental Figure 1. Histological analysis of wild-type aged kidneys subject to ischemic reperfusion injury (IRI). Representative images of aged kidneys 4, 24, 60 days after 45min IRI by periodic acid-Schiff (PAS) staining. Yellow arrow heads indicate the localization of tertiary lymphoid tissues (TLTs). Scale bars: 300 μm.

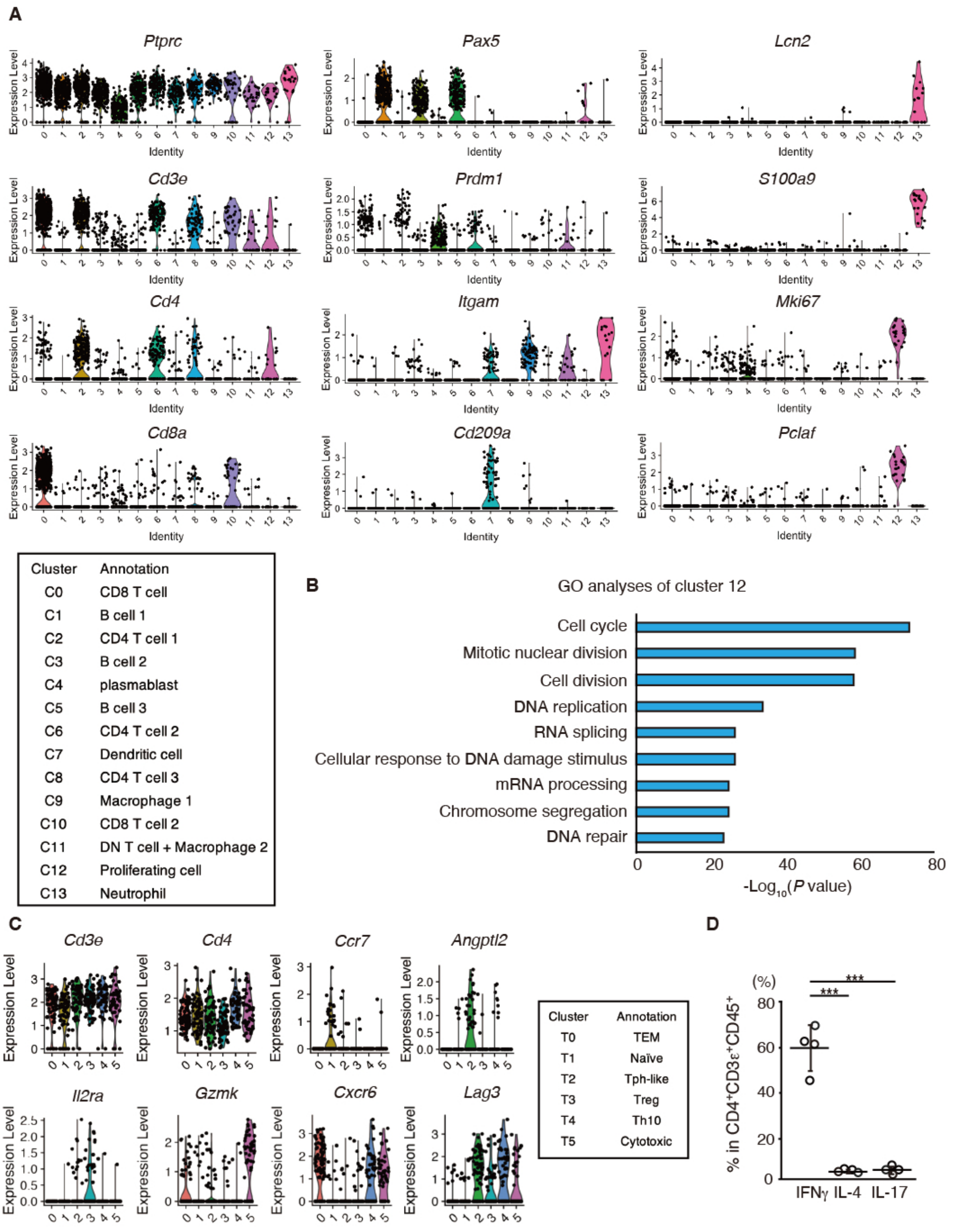

Supplemental Figure 2. Immune cell profiling in aged injured kidneys. (A) Violin plots showing the expression of canonical marker genes across all 14 subsets of $CD45^+$ cells. Fourteen clusters of $CD45^+$ cells were identified (C0-C13) and their putative identities were specified in the box. **(B)** Gene ontology enrichment analysis of upregulated genes in cluster 12. The x axis denotes the -log10 of enrichment *P* values by modified Fisher' s exact test. **(C)** Violin plots showing the expressions of *Cd3ε, Cd4, Ccr7, Angptl2, Il2ra, Gzmk, Cxcr6*, and *Lag3* in six clusters of $CD4^+$ T cells in aged injured kidneys 45 days after ischemic reperfusion (IRI). The putative identities of each cluster were specified in the box. **(D)** IFNγ, IL-4 and IL-17 production by $CD4^+$ T cells from aged injured kidneys, represented as the percentage of positive cells (n = 4/group). Cells were stimulated in bulk culture with PMA and ionomycin. Values are shown as means ± SD, and statistical significance was determined by one-way ANOVA with Bonferroni post hoc analysis. ***$P < 0.001$.

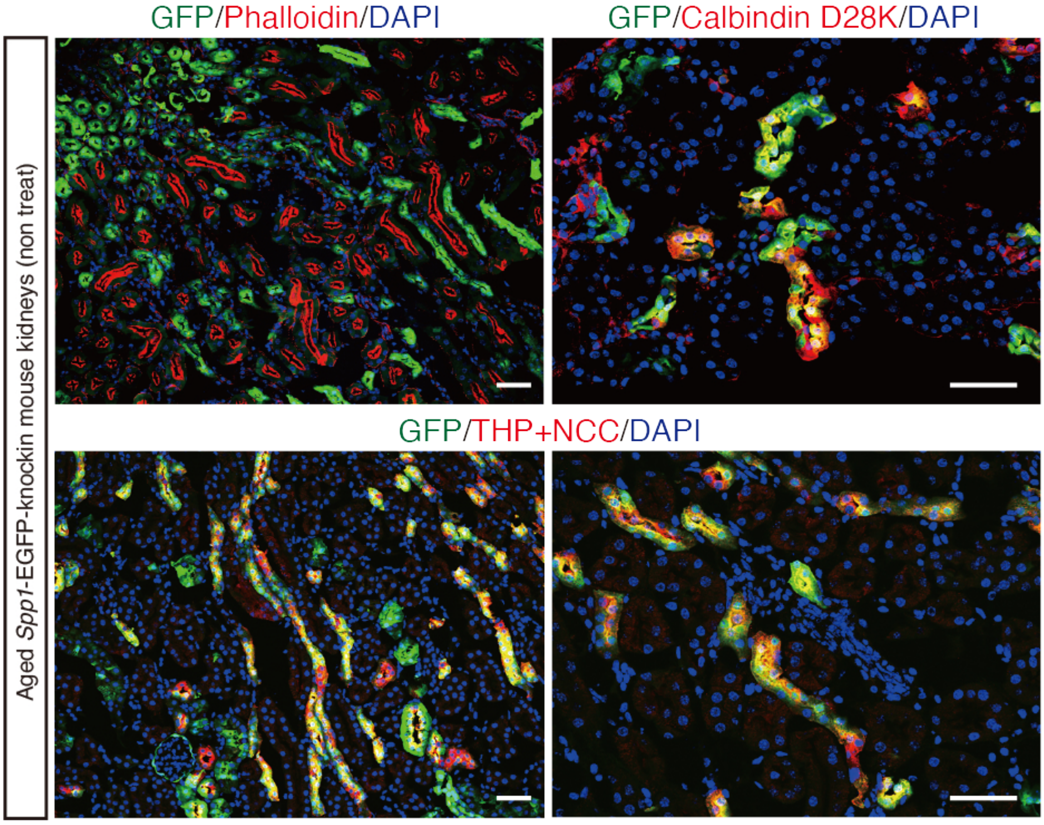

Supplemental Figure 3. *Spp1* are expressed in part of the distal tubules in the kidneys under physiological condition. Immunofluorescence of GFP and phalloidin (a marker for proximal tubules), Tamm-Horsfall protein (THP) (a marker for thick ascending limbs) and thiazide-sensitive sodium chloride co-transporter (NCC) (a marker for distal convoluted tubules), and calbindin D28K (a marker for distal convoluted tubules) in the kidneys of aged *Spp1*-EGFP-knockin mice under physiological condition. EGFP was visualized with an anti-GFP antibody. Scale bar: 50 μm.

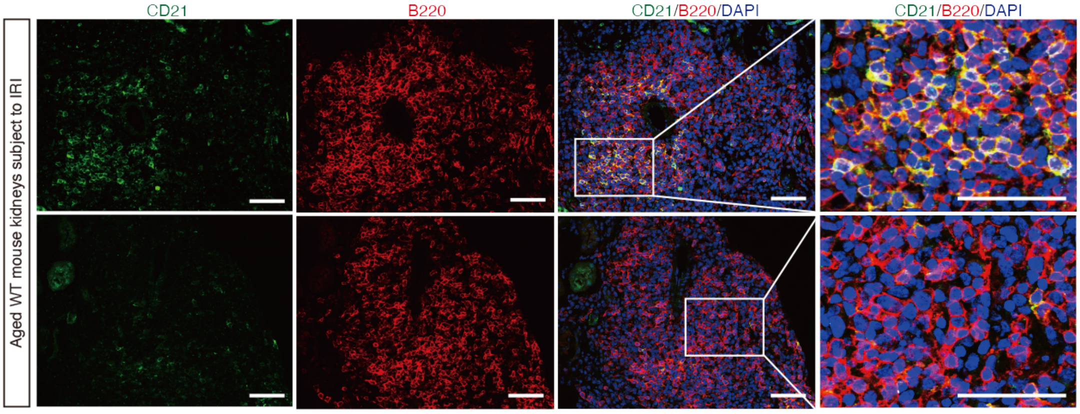

Supplemental Figure 4. Both CD21⁺ and CD21⁻ B cells exist within TLTs. Immunofluorescence of CD21 and B220 in aged injured kidneys 45 days after ischemic reperfusion injury (IRI) induction. A magnified view of the outlined box on the left is shown on the right. Scale bars: 50 μm. WT: wild type.

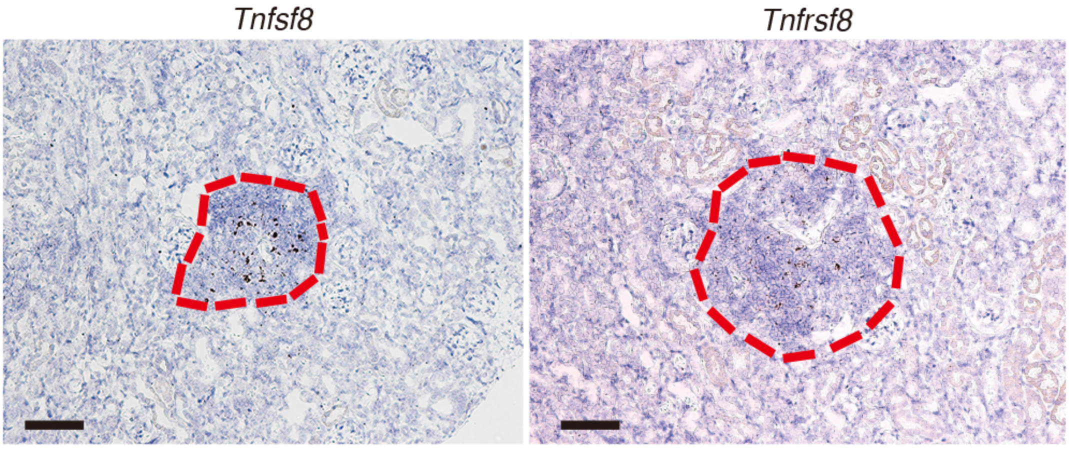

Supplemental Figure 5. *Tnfsf8* and *Tnfrsf8* mRNA expression are almost confined within TLTs in aged injured kidneys. In situ hybridization of *Tnfsf8* (gene name of CD153) and *Tnfrsf8* (gene name of CD30) in aged kidneys 24 days after ischemic reperfusion injury (IRI) induction. The red dotted lines indicate the localization of TLTs. Scale bars: 100μm.

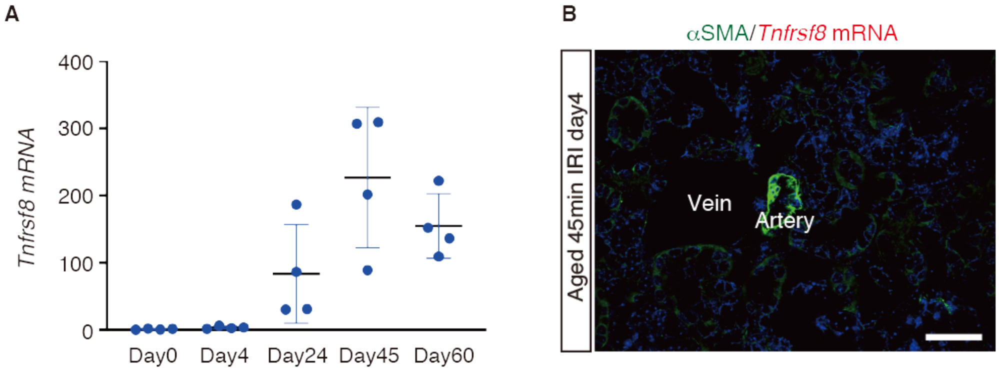

Supplemental Figure 6. *Tnfrsf8* mRNA expression are almost undetectable before TLT development in aged injured kidneys. **(A)** Expression levels of *Tnfsf8* (gene name of CD30) mRNA (n = 4/time point) in aged mouse kidneys following ischemic reperfusion injury (IRI). **(B)** Representative in situ hybridization of *Tnfrsf8* with immunofluorescent co-staining for αSMA in aged kidneys 4 days after IRI induction. Scale bar: 50μm.

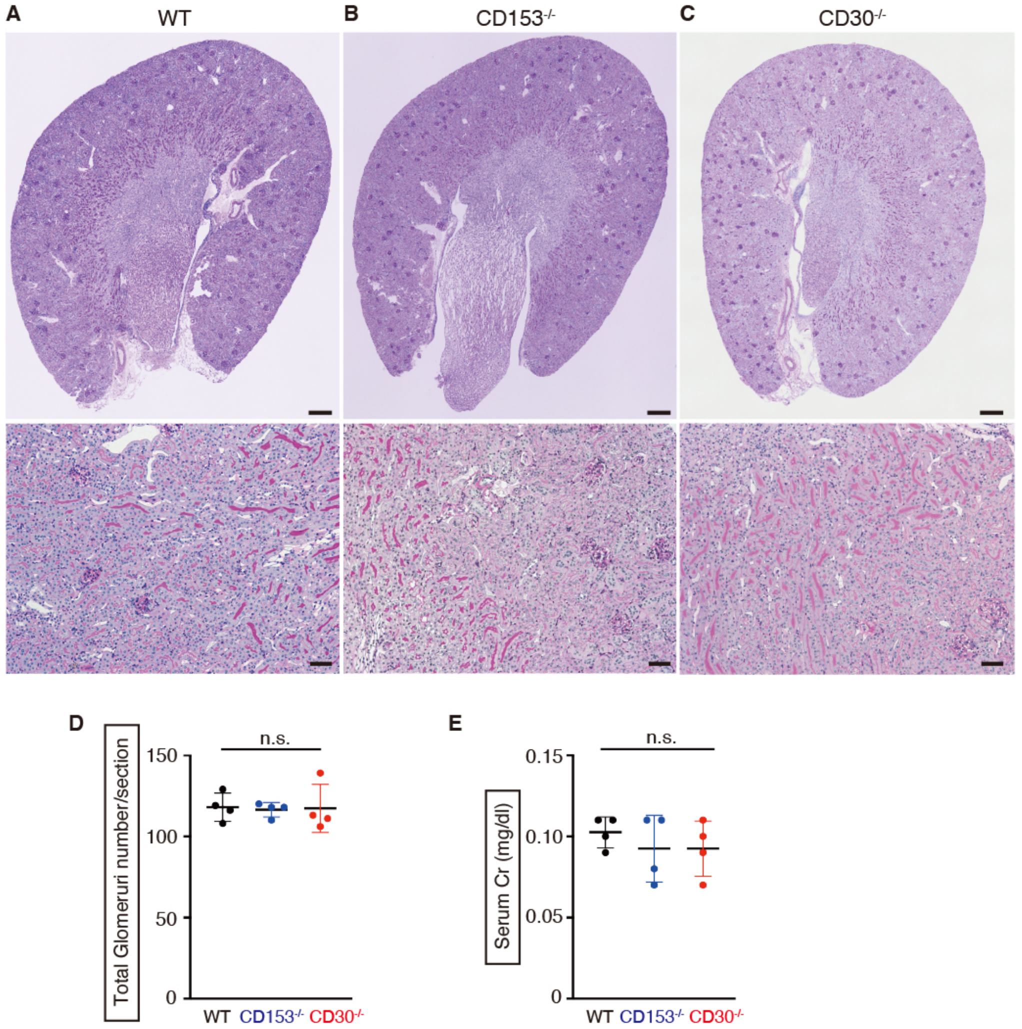

Supplemental Figure 7. Aged *CD153*$^{-/-}$ and *CD30*$^{-/-}$ mice exhibit normal renal function and structure under physiological condition. **(A-C)** Histology (Periodic acid-Schiff (PAS) staining), **(D)** total glomeruli number per section and **(E)** renal function in aged wild-type (WT), CD153-deficient (*CD153*$^{-/-}$) and CD30-deficient (*CD30*$^{-/-}$) mice under physiological condition (n = 4/group). Values are shown as means ± SD, and statistical significance was determined by one-way ANOVA. Scale bars: upper panels 300 μm, lower panels: 50 μm.

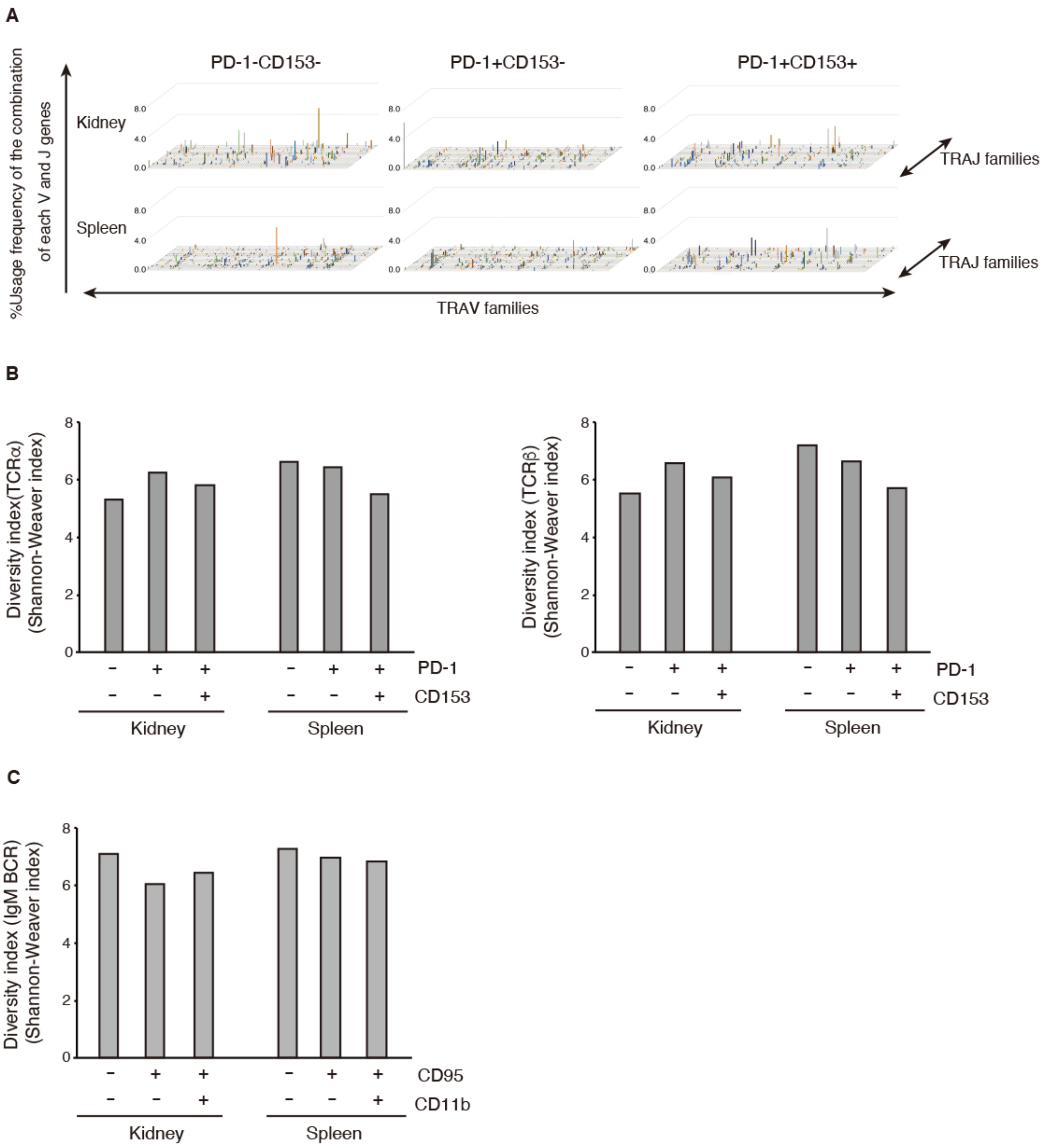

Supplemental Figure 8. Repertoire analysis of CD4⁺ T cells and B cells in aged injured kidneys and spleens. **(A)** T cell receptor (TCR)α repertoire clonalities of three CD4⁺ T cell subpopulations in aged injured kidneys and spleen. The x and y axes indicate the combination of V and J genes (TCR α chain V (TRAV) and TRAJ families), and the z axis indicates their frequencies of usage. **(B-C)** Diversity index (Shannon-Weaver index) of **(B)** TCRα and TCRβ of three CD4⁺ T cell subpopulations and **(C)** IgM B cell receptor (BCR) of three B cell subpopulations in aged kidneys and spleens 45 days after ischemic reperfusion injury (IRI) induction.

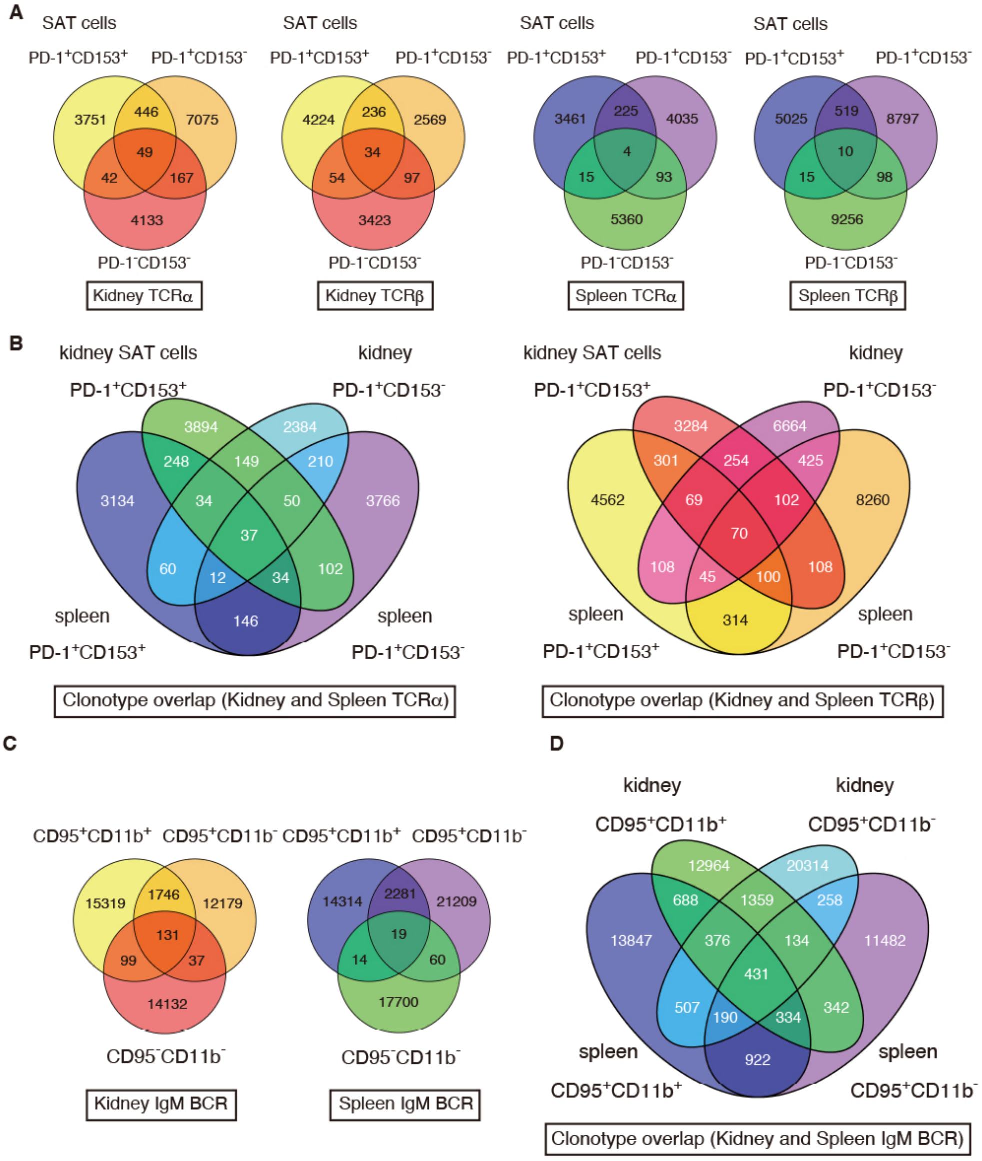

Supplemental Figure 9. Absolute numbers of T cell receptor (TCR) and B cell receptor (BCR) clone in aged injured kidneys and spleens. (A-B) Venn diagram depicting the absolute number of TCRα and TCRβ clonotype shared across **(A)** three CD4^{+} T cell populations and shared across **(B)** PD1^{+}CD153^{-} and PD1^{+}CD153^{+} CD4^{+} T cell populations in aged kidneys and the spleen 45 days after ischemic reperfusion injury (IRI) induction. **(C-D)** Venn diagram depicting the absolute number of IgM BCR clonotype shared across **(C)** three B cell subpopulations within the same organs and across **(D)** CD95^{+}CD11b^{+} and CD95^{+}CD11b^{-} B cell populations in aged kidneys and the spleens 45 days after IRI induction.

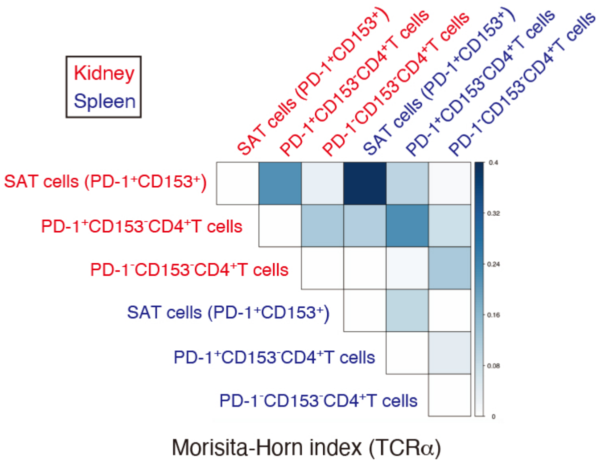

Supplemental Figure 10. Clone sharing between SAT cells and other CD4+ T cells in aged injured kidney and spleen. Heatmap of similarity index (Morishita-Horn index) of T cell receptor (TCR)α clonotype of three CD4+ T cell populations in aged injured kidneys and the spleen 45 days after ischemic reperfusion injury (IRI) induction.

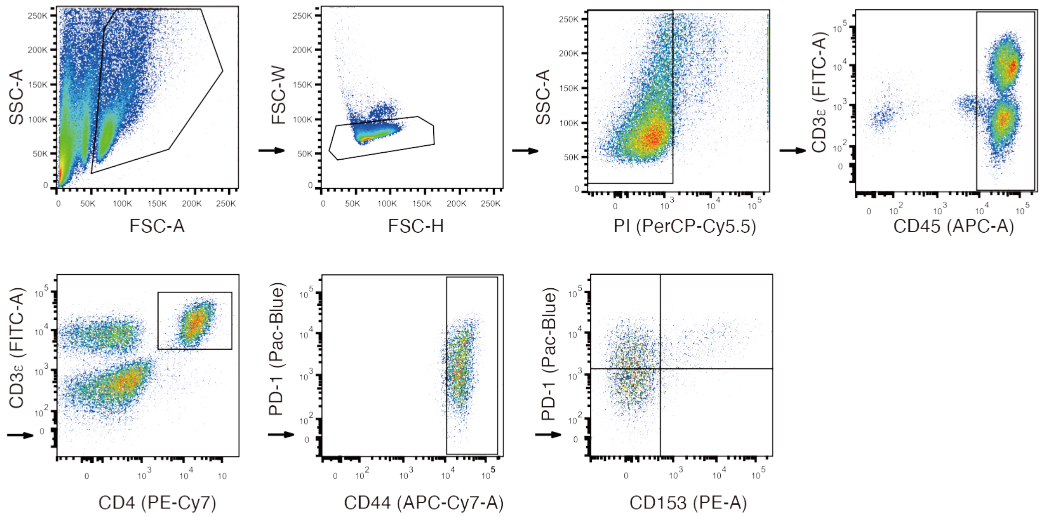

Supplemental Figure 11. Gating strategy to define senescent-associated T (SAT) cells. Representative gating strategy of SAT cells from aged injured kidneys 45 days after ischemic reperfusion injury (IRI) induction. Lymphocytes were gated according to their FSC-A/SSC-A profile to exclude debris, and then doublets were also excluded by the FSC-W/FSC-H profile. Subsequently, single cells were gated on live cells and then gated on $CD45^{+}$ cells. To define SAT cells, $CD45^{+}$ cells were gated on $CD3\varepsilon^{+}CD4^{+}$, followed by $CD44^{high}$ populations, and then $PD\text{-}1^{+}CD153^{+}$ T cells were plotted.

Supplemental Table 1. Primer sequences used for real-time PCR.

Gene	Forward	Reverse
Gapdh	ACGGCAAATTCAACGGCACAGTCA	TGGGGGCATCGGCAGAAGG
Cxcl13	CGTGCCAAATGGTTACAAAGATT	GTGGCTTCAGGCAGCTCTTC
Ccl19	CCTGGGAACATCGTGAAAGC	TGGAGGTGCACAGAGCTGATA
Ifng	CTCATGGCTGTTTCTGGCTGTTAC	TTTCTTCCACATCTATGCCACTTG
Tnfa	CCTCCCTCTCATCAGTTCTATGG	CGTGGGCTACAGGCTTGTC
Col1a1	GTTTGGAGAGAGCATGACCGA	TGGACATTAGGCGCAGGAA
Acta2	AGCGTGAGATTGTCCGTGACA	GCGTTCGTTTCCAATGGTGAT
Il21	TCATCATTGACCTCGTGGCCC	ATCGTACTTCTCCACTTGCAATCCC
Tnfrsf8	GTCCACGGGAACACCATTT	CCAACCAGTAGCACCACCAT
Cd4	AAGGGTTCAGGACAGCGACT	AACTCCGCTGACTCTCCCTC
Cd19	GTCTCTTCTGCTTCTGCCCCAA	GTTCTCAACAGCCAGAGCCACAC
